# Magnetic and Crystal Symmetry Control on Spin Hall Conductivity in Altermagnets

**DOI:** 10.1002/advs.202515002

**Published:** 2025-12-12

**Authors:** Dameul Jeong, Seoung‐Hun Kang, Young‐Kyun Kwon

**Affiliations:** ^1^ Department of Physics and Research Institute for Basic Sciences Kyung Hee University Seoul 02447 South Korea; ^2^ Department of Information Display Kyung Hee University Seoul 02447 South Korea; ^3^ Research Center for Technology Commercialization Korea Institute of Science and Technology Information (KISTI) Seoul 02456 South Korea

**Keywords:** altermagnetism, magnetic symmetry, unconventional spin hall conductivity

## Abstract

Altermagnets combine zero net magnetization with spin splitting, opening new opportunities for next‐generation spintronic devices. In this work, the unconventional spin Hall conductivity (USHC) in three representative materials—RuO_2_, CrSb, and MnTe is explored. It is clarified how distinct magnetic and crystal symmetries modulate their spin Hall responses. RuO_2_ exhibits only trivial USHC under a tilted geometry, demonstrating that symmetry projections alone can induce apparent unconventional elements. In contrast, CrSb and MnTe manifest robust intrinsic USHC driven by symmetry reduction through easy‐axis magnetic ordering without structural tilts. Through extensive first‐principles calculations, the complementary roles of the time‐reversal (T)‐even and T‐odd components in determining the overall spin Hall conductivity are demonstrated. The findings indicate that the interplay between crystal and magnetic symmetry can be controlled through structural tilting and magnetic axis orientation. These results pave the way for the engineering of multifunctional spintronic devices. In particular, they highlight zero‐net‐moment materials with tunable spin configurations as promising platforms for coherent and robust spin transport.

## Graphical abstract

Crystal structures and schematic illustrations of the spin Hall effect (SHE) in altermagnets. High‐symmetry RuO_2_ exhibits conventional SHE, while tilted RuO_2_ shows trivial unconventional SHE (USHE) caused by structural tilting. CrSb and MnTe, with reduced magnetic symmetry, display intrinsic USHE arising from symmetry‐driven spin–momentum locking, leading to nonorthogonal spin‐current geometries.

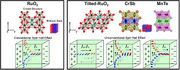

## 1 Introduction

Magnetic materials are conventionally classified as ferromagnets or antiferromagnets. Ferromagnets feature spin‐split conduction bands and large net magnetic moments, while antiferromagnets exhibit no net magnetization through sublattice cancellation.^[^ ^1^, ^2^, ^3^, ^4^ ^]^ However, a new class of magnetism termed altermagnetism has lately attracted attention. It combines the zero‐net‐moment property of antiferromagnets with a rotational‐symmetry‐enforced spin splitting within the unit cell. This concept was first proposed in the original works^[^ ^5^ ^]^ and later consolidated by an editorial classification that emphasized its distinct phenomenology relative to FM and AFM.^[^ ^6^ ^]^ More recent studies have further exemplified symmetry‐driven spin splitting in such systems.^[^ ^7^ ^]^ Comprehensive perspective articles further map out candidate crystals, symmetry diagnostics, and predicted transport responses, positioning altermagnets as fertile ground for spintronics.^[^ ^8^ ^]^

These attributes suggest unusual spin‐transport phenomena that can be harnessed without stray fields. A paradigmatic case is RuO_2_, where a large spin Hall effects have been reported despite weak spin–orbit coupling. Tilted spin currents have been observed experimentally in this material, demonstrating its capability to generate unconventional spin Hall signals in a collinear antiferromagnetic state.^[^ ^9^ ^]^ Subsequent measurements confirmed efficient spin‐to‐charge conversion via altermagnetic spin splitting, highlighting the technological potential of RuO_2_ for spintronic applications.^[^ ^10^ ^]^ Furthermore, theoretical analyses have established the general principle of electrical spin splitting in nonrelativistic collinear antiferromagnets, providing a microscopic basis for these experimental observations.^[^ ^11^ ^]^ Together, these findings underscore the pivotal role of exchange‐driven band topology and Berry curvature in determining spin Hall conductivity (SHC) and motivate a symmetry‐first approach to its analysis in altermagnets, which we pursue below. Indeed, robust SHC in altermagnets suggests the possibility of enhanced coherence time for spins and energy‐efficient spin manipulation because the net magnetic moment is absent, and thus external dipolar fields are diminished.

Beyond conventional SHC (CSHC), altermagnets can exhibit an unconventional SHC (USHC) whenever the spin current, spin polarization, and charge current lose their typical mutual orthogonality.^[^ ^9^, ^12^, ^13^ ^]^ Although trivial USHC can arise from mere coordinate tilting, genuine (or symmetry‐driven) USHC occurs when the magnetic space group forbids conventional orthogonal spin‐current‐charge alignments. This extra degree of freedom can enrich the device design by allowing spin–charge interconversion in geometries not realizable in ordinary ferromagnets or non‐magnetic semiconductors. Previous studies have addressed USHC from complementary perspectives. Roy et al.^[^ ^12^ ^]^ analyzed symmetry‐allowed tensor components in nonmagnetic systems, whereas Mook et al.^[^ ^14^ ^]^ established magnetic‐symmetry rules and microscopic origin of the magnetic spin Hall effect. However, these approaches have remained distinct, and a unified framework that consistently treats both T‐even and T‐odd components under crystal and magnetic symmetries has not been formulated. Here, we extend the symmetry classification of USHC to magnetic systems, providing a unified description that bridges conventional and magnetic spin Hall effects.

In this work, we systematically address how magnetic and crystal symmetries dictate the SHC in three representative systems: the debated candidate RuO_2_ and the established altermagnets CrSb and MnTe. We employ first‐principles of density functional theory (DFT) calculations incorporating Spin–orbit coupling (SOC) and Hubbard corrections, along with the Kubo formalism,^[^ ^14^, ^15^, ^16^, ^17^, ^18^, ^19^, ^20^, ^21^ ^]^ to examine how time‐reversal (T)‐even (Fermi sea) and T‐odd (Fermi surface) terms reshape the SHC in varying symmetry settings. Our main findings are summarized as follows. We establish a framework based on the separation into T‐even and T‐odd contributions, which enables a clear distinction between trivial USHC, as exemplified by tilted RuO_2_, and intrinsic USHC, as realized in CrSb and MnTe. Moreover, we extend conventional crystal‐symmetry analyses of spin Hall conductivity to explicitly incorporate magnetic symmetries, thereby identifying which SHC tensor components remain allowed or are suppressed once the magnetic order is included. A direct comparison of CrSb and MnTe further reveals that, despite sharing the same crystal symmetry, their distinct magnetic easy‐axis orientations lead to different sets of T‐odd USHC elements, underscoring the critical role of magnetic symmetry. Finally, we link these theoretical insights to experimentally accessible signatures—such as sign reversals and anisotropy patterns in spin Hall signals—that may be probed in transport geometries, including spin–orbit torque setups, where the absence of net magnetization minimizes stray‐field effects.

## 2 Computational Methodology

### 2.1 DFT Calculations

Our theoretical framework is based on DFT^[^ ^22^, ^23^ ^]^ implemented in Quantum Espresso.^[^ ^24^, ^25^ ^]^ Projector Augmented Wave pseudopotentials^[^ ^26^ ^]^ were adopted, with an energy cutoff of 60 Ry for wavefunctions and the PBE‐GGA exchange–correlation functional.^[^ ^27^ ^]^ The Brillouin‐zone integrals were performed using Monkhorst–Pack grids of 16 × 16 × 16, 15 × 15 × 9, and 15 × 15 × 7 *k*‐points for RuO_2_, CrSb, and MnTe, respectively. SOC was included self‐consistently, and an additional Hubbard *U* term was introduced for Ru 4*d* (2 eV) and Mn 3*d* (4 eV) orbitals to capture on‐site correlations.^[^ ^5^, ^9^, ^28^, ^29^, ^30^, ^31^, ^32^ ^]^ The adopted values, *U* = 2 eV for Ru^[^ ^5^, ^9^ ^]^ and *U* = 4 eV for Mn,^[^ ^33^, ^34^ ^]^ follow previous DFT+*U* studies on 4*d* ruthenates and 3*d* manganese chalcogenides that successfully reproduced experimental magnetic moments and band structures. We further verified that moderate variations of *U* (by ±1 eV) do not alter the relative magnetic stability or the overall trends in spin Hall conductivity, confirming the robustness of our results.

Following ground‐state computations, we used spinor based maximally localized Wannier functions^[^ ^35^, ^36^ ^]^ to construct tight‐binding Hamiltonians for precise interpolation of band structures and spin transport quantities. The initial projections were chosen as *s*, *p*, and *d*‐orbitals for Ru, Cr, and Mn, and *p*‐orbitals for O, Sb, and Te. The frozen‐window upper bound was set to ≈3 eV above the Fermi level. The SHC was calculated within WannierBerri^[^ ^37^ ^]^ on a dense 100 × 100 × 100 *k*‐mesh and a Fermi level broadening Γ≈50 meV. Details of the k‐mesh convergence test for the SHC are provided in Note S1 (Supporting Information). For consistency, we also checked the SHC results using the Wannier‐based linear response code,^[^ ^16^ ^]^ which confirmed the robustness of our studies.

### 2.2 Spin Hall Conductivity Formalism

SHC is a key parameter for spintronics systems, which facilitates spin‐to‐spin and spin‐to‐charge interconversion. Theoretical estimations of SHC rely on the Kubo formalism, as refined in previous work, to explicitly incorporate material symmetries.^[^ ^14^, ^15^, ^16^, ^17^, ^18^, ^19^, ^20^, ^21^ ^]^ Within this framework, the SHC emerges from two primary contributions, the T‐odd (Fermi surface) and T‐even (Fermi sea) terms, each sensitive to different material symmetries. The T‐odd term applies predominantly to magnetic systems and focuses on states near the Fermi level. Formally, it is defined by

(1) $$\begin{array}{ccc} & & \sigma_{ij}^{odd,k}=\frac{-e\hbar }{2\pi }\Gamma^{2}\int_{BZ}\frac{d^{3}k}{{\left(2\pi \right)}^{3}}\sum_{n,m}\frac{\text{Re}(\left\langle nk|\frac{1}{2}\left\{{\hat{s}}_{k},{\hat{v}}_{i}\right\}|mk\right\rangle \left\langle mk|{\hat{v}}_{j}|nk\right\rangle )}{\left[{\left(E_{F}-\varepsilon_{nk}\right)}^{2}+\Gamma^{2}\right]\left[{\left(E_{F}-\varepsilon_{mk}\right)}^{2}+\Gamma^{2}\right]} \end{array}$$

where ${\hat{s}}_{k}$, ${\hat{v}}_{i,j}$, *E*_F_, ϵ_*n* **k**_ denote the spin and velocity operators, the Fermi level, and the energy of band *n* at **k**, respectively. Γ represents the band broadening constant. In this notation, the three indices *i*, *j*, and *k* of the SHC tensor $\sigma_{ij}^{k}$ represent the directions of the electric field (*j*), the spin–current flow (*i*), and the spin polarization (*k*), respectively. A positive value of $\sigma_{ij}^{k}$ corresponds to spin accumulation with polarization along +*k* on the side transverse to an electric field applied along +*j*, while a negative value indicates the opposite orientation. For the T‐odd component in Equation (1), the conductivity changes sign when the spin‐polarization direction is reversed, consistent with the definition of the spin–current operator $\frac{1}{2}\left\{{\hat{s}}_{k},{\hat{v}}_{i}\right\}$. This odd symmetry under magnetization reversal highlights its importance in systems where the SHC is governed by magnetic symmetry. In contrast, the T‐even contribution is relevant primarily in non‐magnetic systems, as it is associated with states deeply embedded in the Fermi sea. The influence of this term on SHC is expressed as

(2) $$\begin{array}{ccc} & & \sigma_{ij}^{even,k}=-e\hbar \\ & & \int_{BZ}\frac{d^{3}k}{{\left(2\pi \right)}^{3}}\sum_{n,m}\left(f_{nk}-f_{mk}\right)\frac{\text{Im}(\left\langle nk|\frac{1}{2}\left\{{\hat{s}}_{k},{\hat{v}}_{i}\right\}|mk\right\rangle \left\langle mk|{\hat{v}}_{j}|nk\right\rangle )}{{\left(\varepsilon_{nk}-\varepsilon_{mk}\right)}^{2}} \end{array}$$

It maintains even symmetry with respect to magnetization reversal. This symmetry enables the Fermi sea term to characterize SHC in non‐magnetic crystals via symmetry properties independent of magnetization.

Through the combined framework of the Fermi surface and sea terms, SHC can be systematically analyzed across a broad range of materials. The Fermi surface term aligns with magnetic symmetry, while the Fermi sea term predominantly reflects non‐magnetic crystal symmetries, allowing for the tailored manipulation of SHC based on intrinsic material symmetries.^[^ ^15^, ^18^, ^20^, ^21^ ^]^

The SHC tensor, represented as a third‐order tensor, comprises 27 components, with indices *i*, *j*, and *k* independently aligned along the *x*, *y*, or *z* axes. CSHC components arise only when *i*, *j*, and *k* are mutually orthogonal; other configurations are defined as USHC terms.

To facilitate the symmetry analysis, we define a reduced operator $S_{ij}^{k}=v_{i}\sigma_{k}v_{j}$,^[^ ^12^ ^]^ where $v_{i}=\frac{1}{\hbar }\frac{\partial H}{\partial k_{i}}$ is the *i*‐th component of the velocity operator and σ_*k*_ denotes the Pauli spin matrices. This operator $S_{ij}^{k}$ retains the essential tensorial structure of the SHC $\sigma_{ij}^{k}$ but allows for direct evaluation under symmetry operations without explicit electronic‐structure calculations. A symmetry operation O acts on $S_{ij}^{k}$ as follows: if the transformed operator is invariant ($OS_{ij}^{k}=S_{ij}^{k}$), the corresponding SHC tensor element is symmetry‐allowed; if it changes sign ($OS_{ij}^{k}=-S_{ij}^{k}$), the element is forbidden. This selection rule is summarized as

(3) $$OS_{ij}^{k}=\left\{\begin{array}{cc} S_{ij}^{k}, & \text{allowed},\\ -S_{ij}^{k}, & \text{disallowed} \end{array}\right.$$

This concise rule, originally formulated for T‐even SHC in non‐magnetic crystals,^[^ ^12^ ^]^ is here extended to magnetic systems by including time‐reversal–related operations relevant to T‐odd SHC. It provides a general criterion for identifying the presence or absence of specific SHC tensor elements directly from symmetry operation, without explicit electronic‐structure calculations. Consequently, while the T‐even component $\sigma_{ij}^{\text{even},k}$ is determined purely by crystal symmetry, the T‐odd component $\sigma_{ij}^{\text{odd},k}$ additionally requires consideration of magnetic symmetries due to its explicit time‐reversal asymmetry. Once the relevant crystal and magnetic symmetries of a material are identified, Equation (3) directly specifies which SHC tensor components are symmetry‐allowed or forbidden, providing a general predictive rule applicable to other systems without explicit first‐principles calculations.

## 3 Results and Discussion

### 3.1 Structural and Spin Orientations in Altermagnets

**Figure 1:**
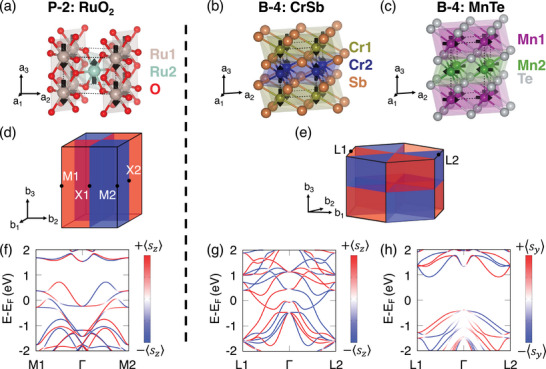
Crystal structures for a) RuO_2_, b) CrSb, and c) MnTe, with lattice vectors **a**_1_, **a**_2_, and **a**_3_ (**a**_1_ along the *x*‐axis). Brillouin zones of d) P‐2 and e) B‐4 symmetries with spin–momentum locking, where reciprocal lattice vectors **b**_1_, **b**_2_, and **b**_3_ (**b**_1_ along *k*_*x*_ in (d) and **b**_2_ along *k*_*y*_ in (e)). Red and blue denote opposite spin–momentum locking directions. Spin–orbit coupling (SOC)‐resolved band structures for f) RuO_2_, g) CrSb, and h) MnTe show spin projections: red/blue lines indicate positive/negative spin projections along *z* (RuO_2_, CrSb) or *y* (MnTe).

RuO_2_, CrSb, and MnTe are recognized altermagnets, each exhibiting unique magnetic properties. Although the magnetic nature of RuO_2_ remains debated,^[^ ^38^, ^39^, ^40^, ^41^, ^42^, ^43^, ^44^, ^45^, ^46^, ^47^ ^]^ in this work, we consider both possibilities: (i) if RuO_2_ is not an altermagnet, as explicitly analyzed in Note S2 (Supporting Information), where we present non‐magnetic calculations (*U* = 0 to 1 eV) showing that T‐odd SHC remains vanishing in this regime. (ii) if RuO_2_ is treated as an altermagnet, following prior studies on tilted crystals.^[^ ^9^ ^]^ For the main text, we adopt the latter assumption in order to compare the trivial USHC (RuO_2_) with the intrinsic USHC (CrSb and MnTe), while making clear that our conclusions remain valid regardless of RuO_2_'s final classification. **Figure** **1**a–c depicts the crystal structures of RuO_2_, CrSb, and MnTe, along with their respective easy axes identified in previous works.^[^ ^48^, ^49^, ^50^, ^51^, ^52^, ^53^, ^54^ ^]^ According to the classification by Šmejkal et al.,^[^ ^5^ ^]^ RuO_2_ is a planar altermagnet (P‐2), while CrSb and MnTe fall into the bulk category (B‐4). Figure 1d,e schematically illustrate spin–momentum locking within the first Brillouin zone for the category. RuO_2_, CrSb, and MnTe demonstrate robust spin splitting despite SOC‐induced perturbations.^[^ ^55^ ^]^ As shown in Figure 1f–h, each compound exhibits clear spin polarization in its band structure, with RuO_2_ and CrSb spins aligned along $\hat{z}$, and MnTe along $\hat{y}$ ($\left[01\bar{1}0\right]$) in our coordinate system. A detailed comparison of band structures with and without SOC is provided in Note S3 (Supporting Information).

### 3.2 Trivial USHC in Tilted RuO_2_

**Figure 2:**
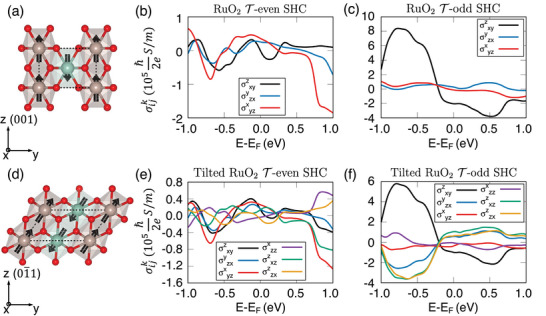
Fermi sea (time‐reversal (T)‐even) and surface (T‐odd) contributions to the spin Hall conductivity (SHC) of RuO_2_ along the (001) and ($0\bar{1}1$) orientations. Side views of RuO_2_ with magnetic moments aligned along [001] for the a) (001) and d) ($0\bar{1}1$) orientations, respectively. b) The sea and c) surface contributions to the SHC for the (001)‐oriented RuO_2_, where symmetry only allows conventional SHC (CSHC) elements. Rotation symmetry allows six CSHC elements, reducible to three independent components. e) The sea and f) surface contributions to SHC for the ($0\bar{1}1$)‐oriented RuO_2_. The tilted orientation and spin alignment break symmetry, introducing unconventional SHC (USHC) elements. However, these contributions are trivial due to identical results from both sea and surface terms.

In RuO_2_, SHC arises from distinct contributions of the Fermi sea and surface, constrained by the 4/*mmm* Laue symmetry in the crystal. **Figure** **2**a shows spin aligned with the *z*‐direction((001) axis), where only CSHC elements contribute from the Fermi sea. Figure 2b,c highlights these CSHC terms corresponding to spin polarizations in *x*, *y*, and *z*. The non‐symmorphic symmetry, involving glide‐reflection symmetry as a combination of mirror symmetry $\left(M_{x,y}\right)$ with half translations (τ_*y* + 1/2, *x* + 1/2_ + τ_*z* + 1/2_) (see the Note S4, Supporting Information), imposes constraints on the components of SHC to be only CSHC.

In contrast, a ($0\bar{1}1$)‐oriented RuO_2_ introduces a structural tilt, as shown in Figure 2d, allowing USHC components. This tilt alters both structural and spin directions, allowing trivial USHC contributions from both the Fermi sea and the surface, as shown in Figure 2e,f. Trivial USHC arises from structural tilts rather than intrinsic magnetic symmetry breaking. The trivial nature of USHC is evidenced by the identical matrix elements derived from both the Fermi sea and surface contributions, regardless of the tilting angle. All SHC tensor elements for tilted structures can be determined analytically with a tilting angle, removing the need for direct DFT calculation.

### 3.3 Symmetry‐Driven Intrinsic USHC in CrSb and MnTe

**Figure 3:**
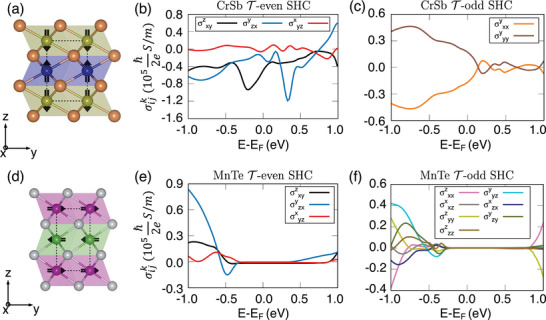
Magnetic symmetry‐Driven USHC Variations in CrSb and MnTe. Side views of a) CrSb with magnetic moments aligned along [0001] and d) MnTe with magnetic moments aligned along $\left[01\bar{1}0\right]$, respectively. b) Fermi sea and c) Fermi surface contributions to the SHC in CrSb; e) Fermi sea and f) Fermi surface contributions to the SHC in MnTe. For both CrSb and MnTe, the Fermi sea contribution permits only CSHC elements. However, when magnetic symmetry is applied to the Fermi surface contribution, CSHC components are disallowed. Although CrSb and MnTe share the same crystal structure, their differing magnetic symmetries, due to distinct, easy axes, lead to variations in the permissible USHC elements. Moreover, MnTe lacks magnetic rotational symmetry, resulting in all USHC elements being independent of each other.

In contrast, CrSb and MnTe display distinct USHC characteristics that separate them from RuO_2_. In **Figure** **3**a,d, the side views illustrate the magnetic moment orientations, where CrSb exhibits moments along [0001], whereas MnTe displays moments along $\left[01\bar{1}0\right]$. As shown in Figure 3b,e, both materials belong to the 6/*mmm* Laue group, which confines T‐even (Fermi‐sea) contributions to CSHC components, resembling the behavior of nonmagnetic systems with the same symmetry group.^[^ ^12^ ^]^ These T‐even contributions are primarily governed by crystal symmetry. On the other hand, as shown in Figure 3c,f, the T‐odd (Fermi‐surface) contributions in CrSb and MnTe diverge from the constraints of CSHC, permitting only a specific subset of USHC tensor elements.

In CrSb, uniaxial magnetization along [0001] breaks certain mirror planes that would otherwise preserve orthogonality. Consequently, spin–orbit coupling lifts degeneracies in the nodal plane *k*_*z*_ = 0, giving rise to strong in‐plane spin textures. The components $\sigma_{xx}^{\text{odd},y}$, $\sigma_{yx}^{\text{odd},x}$, and $\sigma_{xy}^{\text{odd},x}$ are equivalent due to constraints imposed by rotational symmetry *C*_3*z*_. Meanwhile, in MnTe, the spins lie in the $\left[01\bar{1}0\right]$ direction, further reducing the effective magnetic symmetry. This yields an even broader set of symmetry‐allowed USHC elements, as illustrated in Figure 3f, each exhibiting distinct magnitudes due to the lack of higher‐order rotational equivalences.

We summarize in **Table** **1** the classification of unconventional spin Hall conductivity (USHC) contributions across RuO_2_, CrSb, and MnTe. The table distinguishes trivial USHC, arising from structural tilts (tilted RuO_2_), from intrinsic USHC, originating from altermagnetic order (CrSb and MnTe), and specifies the corresponding symmetry‐allowed tensor elements. The fundamental difference between these two types of USHC lies in the microscopic role of SOC, which couples to the exchange field associated with magnetic ordering and lowers the crystal symmetry to a distinct magnetic symmetry. This symmetry reduction allows additional SHC tensor components that are otherwise forbidden in the nonmagnetic limit. We examine this SOC–magnetism–symmetry interplay in more detail in the following section.

**Table 1:** Classification of unconventional spin Hall conductivity (USHC) contributions in representative altermagnets. The table distinguishes trivial USHC, arising from structural tilts (tilted RuO_2_), from intrinsic USHC, originating from altermagnetic order (CrSb and MnTe), and lists their corresponding physical origins and symmetry‐allowed tensor elements.

Material	Type	Physical origin	Symmetry‐allowed USHC elements
RuO_2_	No USHC	Absent mechanism	T‐even = T‐odd; forbidden
Tilted RuO_2_	Trivial USHC	Structural tilt	T‐even = T‐odd; symmetry‐allowed under tilt
CrSb	Intrinsic USHC	Altermagnetic order	T‐even ≠ T‐odd; T‐even: 0, T‐odd: 4
MnTe	Intrinsic USHC	Altermagnetic order	T‐even ≠ T‐odd; T‐even: 0, T‐odd: 7

### 3.4 Band Structures and Spin Textures

**Figure 4:**
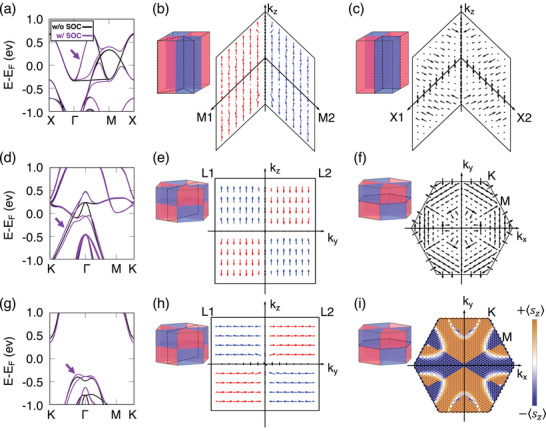
Band structure on the (*k*_*x*_, *k*_*y*_, *k*_*z*_ = 0) plane and spin texture in the Brillouin zone for RuO_2_, CrSb, and MnTe. The spin texture corresponds to the band indicated by the purple arrow in each band‐structure panel. In all spin‐texture plots, the arrow length represents the magnitude of the in‐plane spin. Red and blue arrows denote opposite altermagnetic spin orientations, while black arrows indicate spin components not following the altermagnetic order. a) Band structure of RuO_2_, which exhibits spin degeneracy along the X–Γ and M–X nodal lines, with altermagnetic spin splitting along Γ–M. b) Spin texture on the plane formed by Γ–M1, Γ–M2, and *k*_*z*_, indicated by two dashed planes in the Brillouin zone (BZ). The black line marks the spin‐degenerate nodal line along *k*_*z*_. c) Spin texture on the plane defined by Γ–X1, Γ–X2, and *k*_*z*_, indicated by two dashed planes in the BZ. d) Band structure of CrSb, in which spin splits along all paths in the (*k*_*x*_, *k*_*y*_, *k*_*z*_ = 0) plane when SOC is included. e) Spin texture on the *k*_*y*_–*k*_*z*_ plane indicated by a dashed plane in the BZ. f) Spin texture on the (*k*_*x*_, *k*_*y*_, *k*_*z*_ = 0) plane indicated by a dashed plane in the BZ, which remains purely in‐plane. g) Band structure of MnTe, in which spin splits along all paths within the (*k*_*x*_, *k*_*y*_, *k*_*z*_ = 0) plane. h) Spin texture on the *k*_*y*_–*k*_*z*_ plane indicated by a dashed plane in the BZ. i) Spin texture on the nodal plane (*k*_*x*_, *k*_*y*_, *k*_*z*_ = 0), indicated by a dashed plane in the BZ, showing an out‐of‐plane spin component. The direction is visualized using a dark‐orange–navy color scale, corresponding to spins along the +*z* and −*z* directions.

**Figure** **4** explores the band structure and spin texture in RuO_2_, CrSb, and MnTe, emphasizing the role of symmetry and SOC. Figure 4a shows the RuO_2_ band structure along the (*k*_*x*_, *k*_*y*_, *k*_*z*_ = 0) plane, highlighting spin splitting along Γ‐M with and without SOC and symmetry‐enforced spin degeneracy at nodal lines. Figure 4b,c (projected from the band indicated by the arrow in Figure 4a) illustrates the spin textures on planes defined by *k*_*z*_ and directions M1, M2 ($\left[\bar{1}10\right]$, [110]) and X1, X2 ([010], [100]), respectively, revealing spin‐momentum locking in the relativistic regime and spin degeneracy along high‐symmetry axes such as Γ‐X1, X2, and Z. Without SOC, the nodal planes remain strictly spin‐degenerate and exhibit no spin textures. When SOC is included, these degeneracies are lifted, and spin textures emerge even along the former nodal planes. In RuO_2_, however, such SOC‐induced textures do not translate into a finite USHC, since symmetry constraints still forbid the relevant tensor components despite the lifted degeneracy.

In contrast, CrSb exhibits bulk spin–momentum locking, as shown in Figure 4e, derived from the band marked in Figure 4d. Under non‐magnetic conditions, CrSb shares mirror symmetry planes with RuO_2_, including (*k*_*x*_ = 0, *k*_*y*_, *k*_*z*_), (*k*_*x*_, *k*_*y*_ = 0, *k*_*z*_), and (*k*_*x*_, *k*_*y*_, *k*_*z*_ = 0). The glide‐reflection symmetry ($M_{y}\left(\tau_{x}+\tau_{z}\right)$) preserves the (*k*_*x*_, *k*_*y*_ = 0, *k*_*z*_) plane, as detailed in Note S4 (Supporting Information). However, magnetic symmetry breaks mirror planes such as (*k*_*x*_ = 0, *k*_*y*_, *k*_*z*_) and (*k*_*x*_, *k*_*y*_, *k*_*z*_ = 0), leading to SOC‐driven spin splitting in the *k*_*z*_ = 0 plane, as reflected in Figure 4d and the in‐plane spin texture in Figure 4f.

MnTe shares the same crystal symmetry as CrSb, but exhibits distinct magnetic symmetry due to spin alignment along the [01$\bar{1}$0] direction. Figure 4h, based on the band marked in Figure 4g, shows spins predominantly aligned along the *y*‐axis. With SOC, spin degeneracy is lifted in the (*k*_*x*_, *k*_*y*_, *k*_*z*_ = 0) plane, causing spin alignment along *k*_*z*_, as seen in Figure 4i. This change in spin direction reduces the symmetry of the system. Moreover, unlike RuO_2_ or CrSb, MnTe lacks the glide‐reflection symmetry ($M_{y}\left(\tau_{x+1/2}+\tau_{z+1/2}\right)$) but retains the mirror symmetry ($M_{z}$), preserving σ_*z*_ under its operation. This reduced symmetry, resulting from the [01$\bar{1}$0] spin orientation, enables MnTe to exhibit a broader range of non‐equivalent USHC tensor elements compared to CrSb.

Analytical symmetry operations confirm the unique SHC components of MnTe. There are two non‐trivial symmetry operations in this system to evaluate SHC tensor elements. The symmetry operation combined with time‐reversal and a 180° rotation about the *x*‐axis $TC_{2x}$ and mirror operation $M_{z}$. Among them, $TC_{2x}$ forbids all CSHC components while allowing diverse USHC elements, including $\sigma_{xx}^{\text{odd},z}$, $\sigma_{xz}^{\text{odd},x}$, $\sigma_{yy}^{\text{odd},z}$, $\sigma_{zx}^{\text{odd},x}$, $\sigma_{zy}^{\text{odd},y}$, and $\sigma_{zz}^{\text{odd},z}$. The spin texture in Figure 4h further illustrates spin alignment along the *y*‐axis, with a *k*_*z*_ component appearing upon inclusion of SOC. This orientation reduces symmetry compared to RuO_2_ and CrSb, leading to a greater diversity of USHC tensor elements. The explicit symmetry transformations and analytical derivations of the allowed SHC tensor elements are provided in Note S5 (Supporting Information), where we detail the action of each magnetic space‐group operator on $S_{ij}^{k}$ and demonstrate step‐by‐step how the corresponding forbidden and allowed components arise.

**Figure 5:**
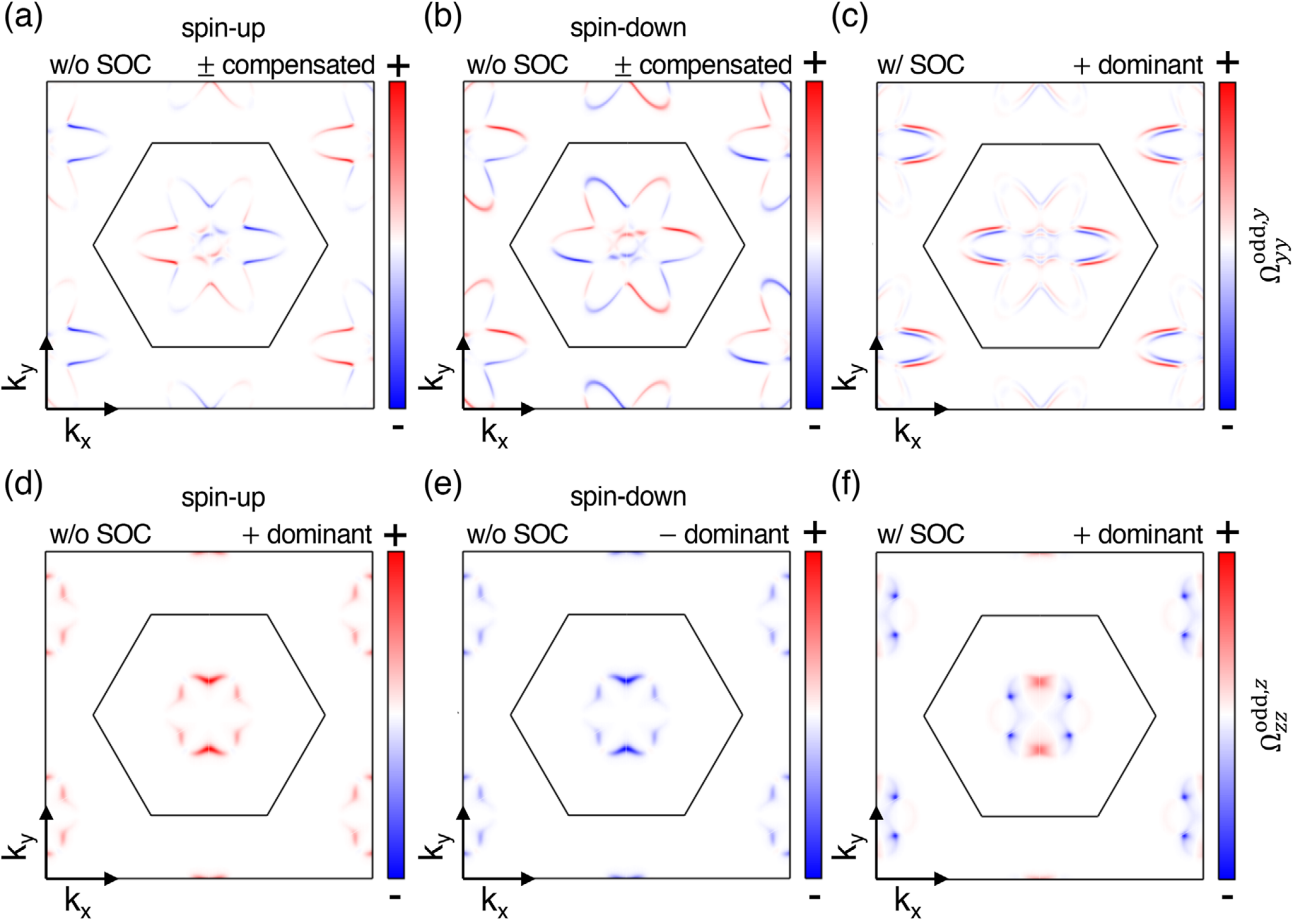
*k*‐resolved T‐odd Berry‐curvature–like unconventional term Ω^odd^ at *E*_F_ −0.5 eV for (*k*_*x*_, *k*_*y*_, *k*_*z*_ = 0) plane with and without SOC. a,b) $\Omega_{yy}^{\text{odd},y}$ of CrSb without SOC for spin‐up and spin‐down channels, and c) with SOC. d,e) $\Omega_{zz}^{\text{odd},z}$ of MnTe without SOC for spin‐up and spin‐down, and f) with SOC. The color scale represents the sign and magnitude of the T‐odd contribution (red = positive, blue = negative; arbitrary units). Without SOC, the spin‐up and spin‐down distributions are opposite in sign and cancel each other, resulting in a vanishing net spin Hall response. When SOC is included, the nodal‐plane degeneracy is lifted, producing finite spin mixing and yielding non‐compensated Ω^odd^ patterns that give rise to a finite USHC.

To directly visualize how SOC lifts the nodal‐plane degeneracy and induces a finite spin Hall response, Figure 5 compares the *k*‐resolved T‐odd Berry‐curvature–like term $\Omega_{ij}^{\text{odd},k}\left(k\right)$ with and without SOC for CrSb ($\Omega_{yy}^{\text{odd},y}$) and MnTe ($\Omega_{zz}^{\text{odd},z}$) at *E*_F_ − 0.5 eV. The Berry‐curvature–like terms are defined as

(4) $$\Omega_{ij}^{\text{odd},k}\left(k\right)=-\Gamma^{2}\sum_{n,m}\frac{\text{Re}(\left\langle n|\frac{1}{2}\left\{{\hat{s}}_{k},{\hat{v}}_{i}\right\}|m\right\rangle \left\langle m|{\hat{v}}_{j}|n\right\rangle )}{\left[{\left(E_{F}-\varepsilon_{n}\right)}^{2}+\Gamma^{2}\right]\left[{\left(E_{F}-\varepsilon_{m}\right)}^{2}+\Gamma^{2}\right]}$$

where ${\hat{s}}_{k}$ and ${\hat{v}}_{i,j}$ denote the spin and velocity operators, respectively, and Γ is the lifetime broadening parameter.

**Figure** **5**a,b shows the $\Omega_{yy}^{\text{odd},y}\left(k\right)$ distribution for CrSb without SOC in the spin‐up and spin‐down channels, while Figure 5c displays the same quantity with SOC. Figure 5d–f presents analogous results for MnTe. Without SOC, CrSb exhibits opposite‐sign regions within each spin channel, leading to an internal cancellation of the SHC contributions, whereas in MnTe the spin‐up and spin‐down channels have overall opposite signs, and their total contributions cancel each other. Upon inclusion of SOC, the nodal‐plane degeneracy is lifted, and spin mixing occurs, generating finite spin polarization and non‐compensated $\Omega_{ij}^{\text{odd},k}\left(k\right)$ patterns that give rise to a finite unconventional SHC. This direct comparison clearly elucidates the microscopic role of SOC in generating the T‐odd spin Hall response through distinct cancellation mechanisms in CrSb and MnTe. A detailed comparison between the T‐even and T‐odd components, together with the discussion of conventional and unconventional tensor elements, is provided in Note S6 (Supporting Information).

### 3.5 Experimental and Device Design Outlook

Although our work is primarily theoretical, there are several tangible routes to verify and utilize these predicted USHC phenomena in altermagnets:

#### 3.5.1 Epitaxial Growth and Strain Control

Strain in RuO_2_ can be effectively controlled by epitaxial growth on TiO_2_.^[^ ^56^, ^57^ ^]^ Similarly, CrSb and MnTe have also been successfully grown on widely utilized substrates such as GaAs and sapphire,^[^ ^52^, ^53^, ^58^, ^59^ ^]^ where substrate‐induced strain plays a critical role in tuning their magnetic anisotropy. Our results suggest that small shifts in the easy‐axis orientation can move the system between distinct USHC regimes. By measuring spin Hall signals via lock‐in detection of spin‐torque ferromagnetic resonance (ST‐FMR) or second‐harmonic Hall, experimentalists can map the evolution of USHC components under systematically varying strain.

#### 3.5.2 Spintronic Device Architectures

Altermagnets offer zero net moment and strong spin–split bands—ideal for spin–orbit torque (SOT) devices. Recent studies of USHC in low‐symmetry systems (e.g., IrO_2_)^[^ ^60^ ^]^ using ST‐FMR have shown that unconventional spin current components can be generated and detected efficiently. Analogous bilayer devices combining heavy metals with CrSb or MnTe could enable current‐driven spin injection and re‐emission along unconventional directions, enabling multifunctional operations controllable by strain or doping.

#### 3.5.3 Transport Readouts and Experimental Probes

To bridge our theoretical predictions with measurable observables, we outline of feasible experimental probes capable of verifying the unconventional spin Hall conductivity (USHC) in altermagnets. Angular‐dependent longitudinal and planar Hall measurements in epitaxial films can serve as indirect probes of symmetry‐encoded USHC anisotropy, since angle‐dependent transport coefficients reflect the underlying crystal and magnetic axes. Such protocols are already well established in antiferromagnets, including MnTe, where anisotropic magnetoresistance has been demonstrated.^[^ ^61^ ^]^ Harmonic Hall analysis in heavy‐metal/altermagnet bilayers can separate damping‐like and field‐like spin–orbit torques and determine their sign, which directly reflects the polarity of the spin Hall current.^[^ ^62^, ^63^ ^]^ This approach provides a direct means to distinguish trivial (tilted RuO_2_) from intrinsic (CrSb, MnTe) USHC contributions. In addition, spin‐torque ferromagnetic resonance (ST‐FMR) can quantify both the sign and magnitude of spin–orbit torques with high precision, offering an independent validation of the same predictions. These experimental protocols are already well established in spin–orbit torque and antiferromagnetic spintronics,^[^ ^64^ ^]^ and thus represent realistic near‐term routes to benchmark our symmetry‐based theoretical framework.

## 4 Conclusion

In this study, we systematically investigated the unconventional spin Hall conductivity (USHC) in representative altermagnetic materials, such as RuO_2_, CrSb, and MnTe, highlighting the critical roles played by their distinct magnetic and crystal symmetries. RuO_2_ was shown to exhibit only trivial USHC contributions induced by structural tilting, without genuine intrinsic unconventional features. In contrast, CrSb and MnTe demonstrated significant intrinsic USHC arising explicitly from their reduced magnetic symmetry. Our results underline how different magnetic easy‐axis orientations, combined with crystal symmetry breaking, strongly influence both time‐reversal‐even (Fermi‐sea) and time‐reversal‐odd (Fermi‐surface) SHC contributions.

These findings open new opportunities for the engineering of advanced spintronic devices using altermagnets. Specifically, we propose experimental pathways, such as epitaxial strain control, doping, and alloying, to manipulate and optimize the magnitude and anisotropy of USHC. Such tunability of spin currents in materials with zero net magnetization provides a significant advantage, reducing undesirable stray fields and enhancing spin coherence.

Ultimately, our work establishes a clear theoretical foundation for the experimental realization of novel altermagnet‐based spintronic applications. Beyond material‐specific implications, our study emphasizes the need to extend interpretations of spin Hall conductivity from crystal to magnetic symmetries. This broader perspective explains the contrasting signs and anisotropies of USHC in RuO_2_, CrSb, and MnTe, and offers a general framework to guide future theoretical analyses and experimental investigations of symmetry‐protected spin transport.

We also note that the ongoing debate surrounding RuO_2_ underscores the need for careful experimental validation of candidate altermagnets. Importantly, this does not undermine our central conclusions; rather, it highlights the utility of our framework for distinguishing trivial from intrinsic USHC across different materials.

Finally, we stress that the symmetry‐based framework developed here is not restricted to centrosymmetric altermagnets. Recent studies^[^ ^65^ ^]^ on chiral metallic dichalcogenides have shown that symmetry‐enforced persistent spin textures can coexist with altermagnetic spin splitting and govern charge‐to‐spin conversion, underscoring the universality of symmetry‐driven spin responses across diverse material classes.

## Conflict of Interest

The authors declare no conflict of interest.

## Supporting information



Supporting Information

## Data Availability

The data that support the findings of this study are available from the corresponding author upon reasonable request.
