# Supplementary material for: Magnetic and Crystal Symmetry Control on Spin Hall Conductivity in Altermagnets
Source: Adv Sci (Weinh). 2025 Dec 12;13(13):e15002. doi: 10.1002/advs.202515002 (PMC12955886; doi:10.1002/advs.202515002)
Supplement: Supplementary file 1 — Supporting Information [file ADVS-13-e15002-s001.pdf]

Supporting Information for

Magnetic and Crystal Symmetry Control on Spin Hall Conductivity in Altermagnets

Dameul Jeong,[1] Seoung-Hun Kang,[1,2,3,*] and Young-Kyun Kwon[1,2,†]

[1]*Department of Physics and Research Institute for Basic Sciences, Kyung Hee University, Seoul, 02447, Korea*

[2]*Department of Information Display, Kyung Hee University, Seoul, 02447, Korea*

[3]*Research Center for Technology Commercialization, Korea Institute of Science and Technology Information (KISTI), Seoul 02456, Korea*

S1. CONVERGENCE TEST FOR SHC

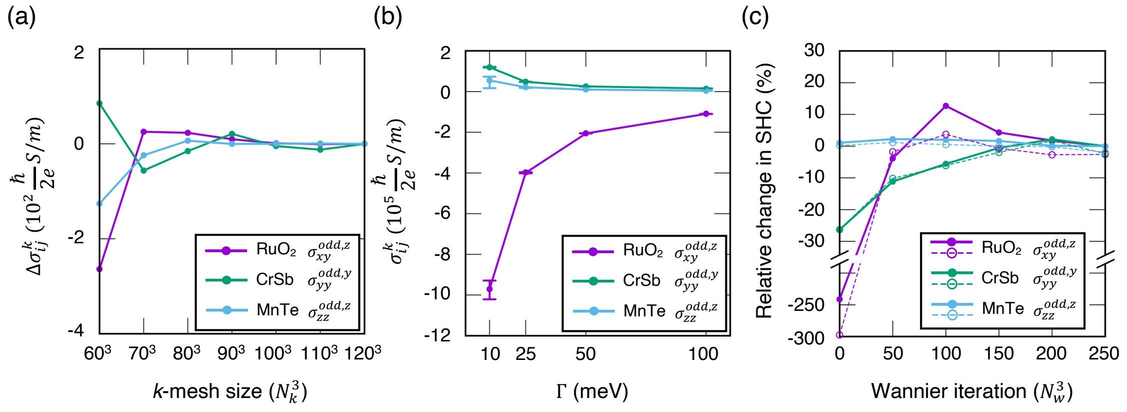

FIG. S1. Convergence and broadening dependence of the $\mathcal{T}$-odd SHC in RuO_2, CrSb, and MnTe. (a) k-mesh convergence test of the SHC. The vertical axis represents the deviation $\Delta\sigma_{ij}^{k}$ with respect to the value obtained using a 120^3 mesh. For RuO_2 (purple, $\sigma_{xy}^{\text{odd},z}$) and CrSb (green, $\sigma_{yy}^{\text{odd},y}$), the SHC is evaluated at the Fermi level, whereas for MnTe (blue, $\sigma_{zz}^{\text{odd},z}$) it is taken 0.76 eV below E_{F} where the SHC reaches its maximum. The results show that the SHC values converge rapidly with increasing k-mesh density and are already well converged at 100^3, confirming the numerical robustness of our calculations. (b) Dependence of the $\mathcal{T}$-odd SHC on the band broadening parameter Γ. The error-bar–like symbols represent the range between the maximum and minimum SHC values obtained for k-meshes from 60^3 to 200^3. The largest numerical uncertainty appears at $\Gamma = 10$ meV, while for $\Gamma \geq 25$ meV the variation becomes negligible, indicating that the results are stable against the choice of Γ. (c) Dependence of the $\mathcal{T}$-odd SHC on Wannierization parameters, namely the number of Wannier iterations (N_{W}) and the upper bound of the frozen-window energy relative to E_{F}. The relative change in SHC is referenced to the value obtained at $N_{\text{W}} = 250$ with a frozen-window upper bound of $E_{\text{F}} + 3$ eV. Solid and dashed lines correspond to frozen-window upper bounds of $E_{\text{F}}+3$ and $E_{\text{F}}+3.5$ eV, respectively. The SHC exhibits larger numerical fluctuations at low iteration numbers ($N_{\text{W}} \leq 100$), but the variation decreases rapidly below 5% for $N_{\text{W}} = 150$ and below 3% for $N_{\text{W}} = 200$. These results indicate that the SHC is sensitive to Wannierization parameters only at low iteration numbers and becomes numerically stable once convergence is achieved ($N_{\text{W}} \geq 150$).

To assess the numerical robustness of our calculations, we performed k-mesh convergence

tests for the $\mathcal{T}$-odd SHC. The deviation relative to the 120^3 grid is defined as $\Delta\sigma_{ij}^k = \sigma_{ij}^k(N_k^3) - \sigma_{ij}^k(120^3)$, where $\sigma_{ij}^k(N_k^3)$ denotes the SHC obtained with an N_k^3 k-mesh. Fig. S1(a) shows the convergence behavior for RuO_2, CrSb, and MnTe. For RuO_2 and CrSb, the SHC is evaluated at the Fermi level, while for MnTe it is taken 0.76 eV below E_F where the SHC reaches its maximum. The results indicate that the SHC converges rapidly with increasing k-mesh density and that a 100^3 grid is sufficient to ensure numerical convergence. Thus, the SHC values reported in the main text are robust against residual errors from k-point sampling.

Fig. S1(b) presents the dependence of the $\mathcal{T}$-odd SHC on the quasiparticle broadening parameter Γ. The error-bar–like symbols represent the range between the maximum and minimum SHC values obtained from k-meshes of 60^3 to 200^3, thereby quantifying the numerical uncertainty associated with k-point sampling. The largest variation occurs at $\Gamma = 10$ meV, whereas for $\Gamma \geq 25$ meV the SHC becomes essentially insensitive to Γ, confirming the stability of the calculated results with respect to both Γ and k-mesh density. Consistent with previous first-principles studies on RuO_2 and related metallic systems—where the physically relevant scattering regime lies between 10 to 100 meV and the $1/\Gamma$ scaling behavior persists up to ~100 meV[S1,S2], we adopt $\Gamma = 50$ meV as a representative value. This choice ensures both numerical robustness and physical relevance for comparison with experimentally accessible conductivities.

Fig. S1(c) further examines the sensitivity of the $\mathcal{T}$-odd SHC to Wannierization parameters, namely the number of Wannier iterations (N_W) and the upper bound of the frozen-window energy relative to E_F. The SHC variation is shown as a relative change with respect to the value obtained at $N_W = 250$ and a frozen-window upper bound of $E_F + 3$ eV. Solid and dashed lines correspond to frozen-window upper bounds of $E_F + 3$ and $E_F + 3.5$ eV, respectively. The SHC exhibits larger numerical fluctuations at low iteration numbers ($N_W \leq 100$), but the deviation rapidly decreases below 5 % for $N_W = 150$ and below 3 % for $N_W = 200$. These results indicate that, similar to k-mesh and Γ sampling, variations in the Wannierization parameters are an important factor for convergence. Once properly converged, however, the SHC values remain numerically stable, confirming that the Wannier fitting is well controlled.

Fig. S2 further presents the full energy dependence of the $\mathcal{T}$-odd spin Hall conductivity σ_{ij}^k for different broadening parameters $\Gamma = 10$, 25, 50, and 100 meV, obtained from 100^3 k-

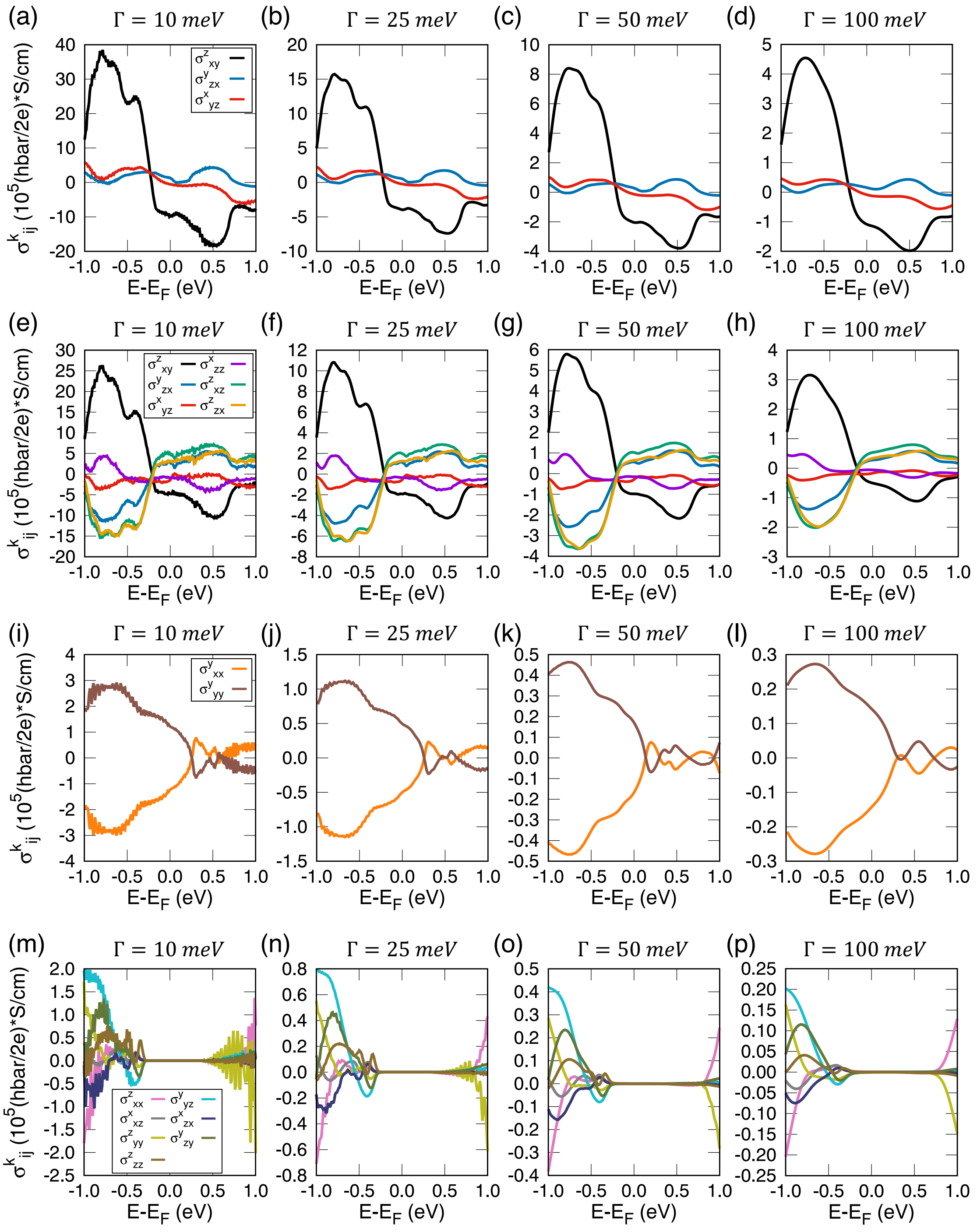

FIG. S2. Energy-dependent $\mathcal{T}$-odd SHC σ^k_{ij} for different band broadening parameters $\Gamma = 10$, 25, 50, and 100 meV. (a–d) RuO_2, (e–h) tilted RuO_2, (i–l) CrSb, and (m–p) MnTe. Each panel shows the evolution of the $\mathcal{T}$-odd SHC spectra with increasing Γ, illustrating the robustness and damping behavior of the spin Hall response against disorder scattering.

mesh calculations for all four systems: (a–d) RuO_2, (e–h) tilted RuO_2, (i–l) CrSb, and (m–p) MnTe. Each panel shows how the spectral shape evolves with increasing Γ, illustrating the robustness of the tensor signs and qualitative trends, while only the absolute magnitudes vary smoothly.

S2. SPIN HALL CONDUCTIVITY OF NON MAGNETIC PHASE RuO_2

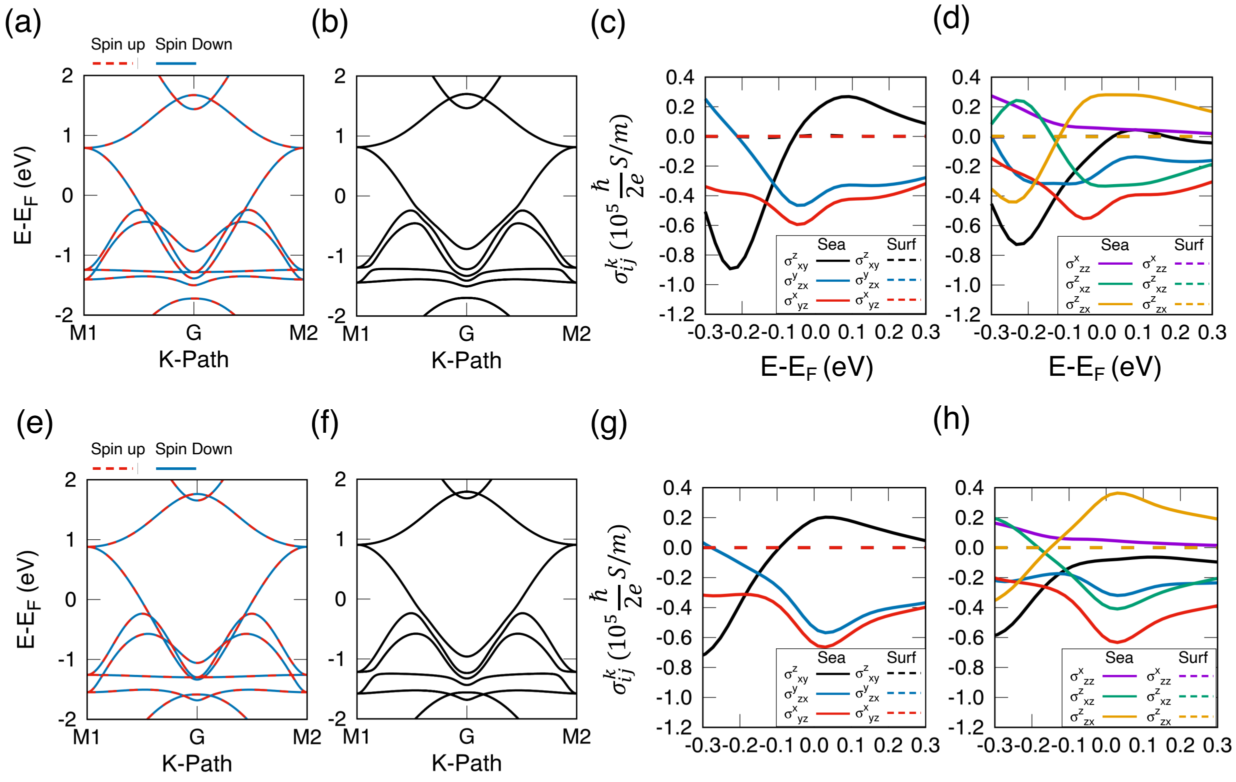

FIG. S3. Band structure and spin Hall conductivity (SHC) of nonmagnetic phase RuO_2 when Hubbard U=0 (a-d), $U = 1.0$ (e-h). (a),(e) Band structure without spin orbit coupling (SOC). The red dotted line is a spin-up, and the blue solid line is a spin-down band. (b),(f) Band structure with SOC. (c),(g) SHC with Fermi sea contribution (solid line) and surface contribution (dotted line) for (001)-oriented RuO_2. (d),(h) SHC with Fermi sea contribution (solid line) and surface contribution (dotted line) for $(0\bar{1}1)$-oriented RuO_2.

There has been an ongoing debate over whether RuO_2 is truly an altermagnet. Neutron diffraction[S3], resonant x-ray scattering[S4,S5], and ARPES experiments[S6] indicate that RuO_2 possesses a non-zero magnetic moment and exhibits altermagnetic behavior. However, Muon spin relaxation studies report a monotonic relaxation of muons within RuO_2[S7], suggesting the absence of an internal magnetic field. Additionally, other neutron diffraction experiments[S8] contradict previous findings, showing no magnetic moment and attributing the antiferromagnetic phase to Ru vacancies. Optical spectroscopy[S9] results support a non-

magnetic band structure, as interband optical measurements align with the nonmagnetic model of RuO_2. ARPES and SARPES experiments[S10] also confirm that RuO_2 matches well with non-magnetic conditions and observe Rashba-like splitting.

To maintain consistency in our analysis, we calculate the band structures and spin Hall conductivity (SHC) of RuO_2 in its nonmagnetic phase. RuO_2 remains nonmagnetic until the effective Hubbard parameter reaches approximately 1.06 eV. In the range between 1.06 and 1.23 eV, it enters a metastable state, and only when the parameter exceeds 1.23 eV does the altermagnetic state become stable[S11]. We implemented the nonmagnetic phase by calculating the cases where the Hubbard U value is 0 and 1. Figs. S3(a) and (b) show the band structure at $U = 0$ without and with SOC, while Figs. S3(e) and (f) display the band structure at $U = 1$. In the nonmagnetic phase, the strong SOC contributes significantly to the Fermi sea contribution on SHC, while the absence of magnetism means there is no Fermi surface contribution as depicted in Figs. S3(c-d) and (g-h). Despite the nonmagnetic phase, the tilted structure still produces unconventional spin Hall conductivity (USHC), similar to an altermagnetic phase shown in Figs. S3 (d) and (f), since the origin of USHC in a tilted structure lies in crystal symmetry. This finding confirms that any material exhibiting the SHC discussed in the main text can also generate USHC in a tilted structure. Thus, the origin of USHC in CrSb and MnTe remains distinct and can be explained even if RuO_2 is not altermagnetic.

S3. ELECTRONIC BAND STRUCTURES

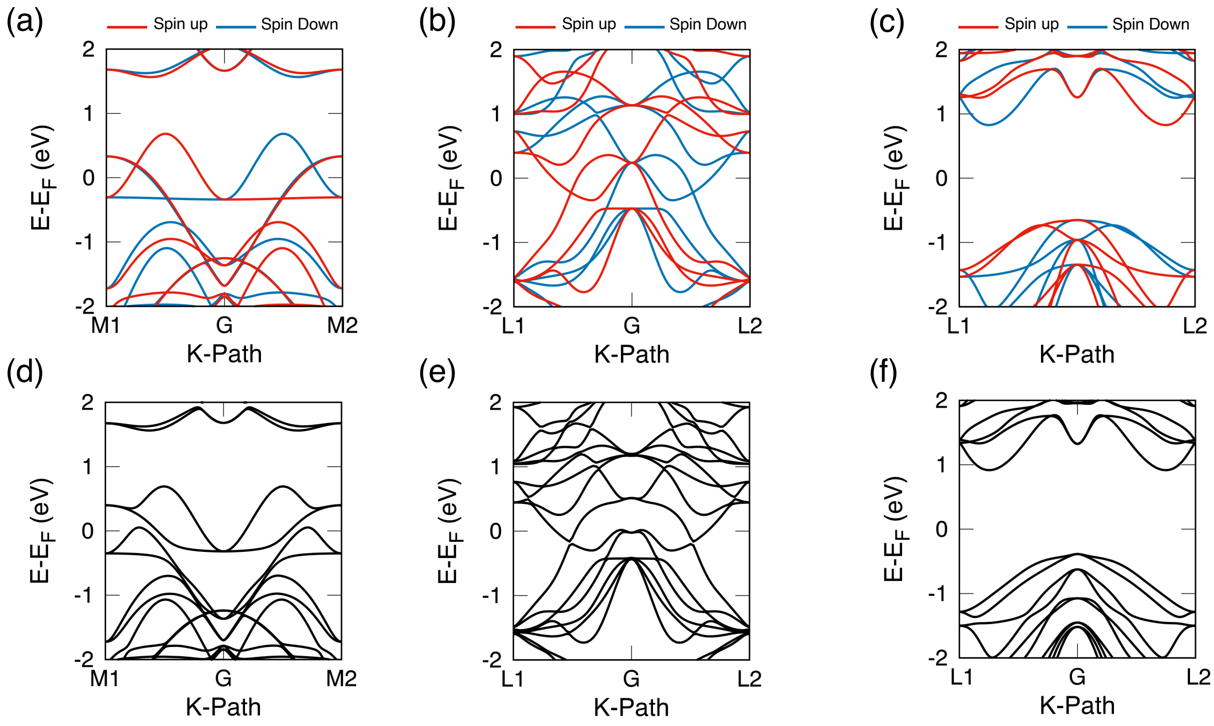

FIG. S4. Band structures of RuO_2, CrSb, and MnTe without SOC (a-c) and with SOC (d-f). The red line is the spin-up band, and the blue line is the spin-down band.

The optimized lattice constants of RuO_2 are $a_1 = a_2 = 4.541$ Å and $a_3 = 3.136$ Å, while those of CrSb and MnTe are $a_1 = a_2 = 4.176$ Å, $a_3 = 5.368$ Å, and $a_1 = a_2 = 4.223$ Å, $a_3 = 6.760$ Å, respectively. The calculated magnetic moments of Ru, Cr, and Mn are 1.133 μ_B, 2.720 μ_B, and 4.573 μ_B, respectively.

Fig. S4 illustrates the electronic band structures of RuO_2, CrSb, and MnTe. Figs. S4(a-c) shows the spin-resolved bands calculated without the spin-orbit coupling (SOC) effect, where the red and blue lines represent the spin-up and spin-down states, respectively. In agreement with previous reports[S2,S12,S13], these systems exhibit altermagnetism with pronounced spin splitting. Figs. S4(d-f) depicts the band structures with SOC included. Despite the significant SOC effects inherent to these materials, the altermagnetic features are preserved, emphasizing the stability of their relativistic electronic structures.

S4. GLIDE-REFLECTION SYMMETRY OF RuO_2 AND CrSb

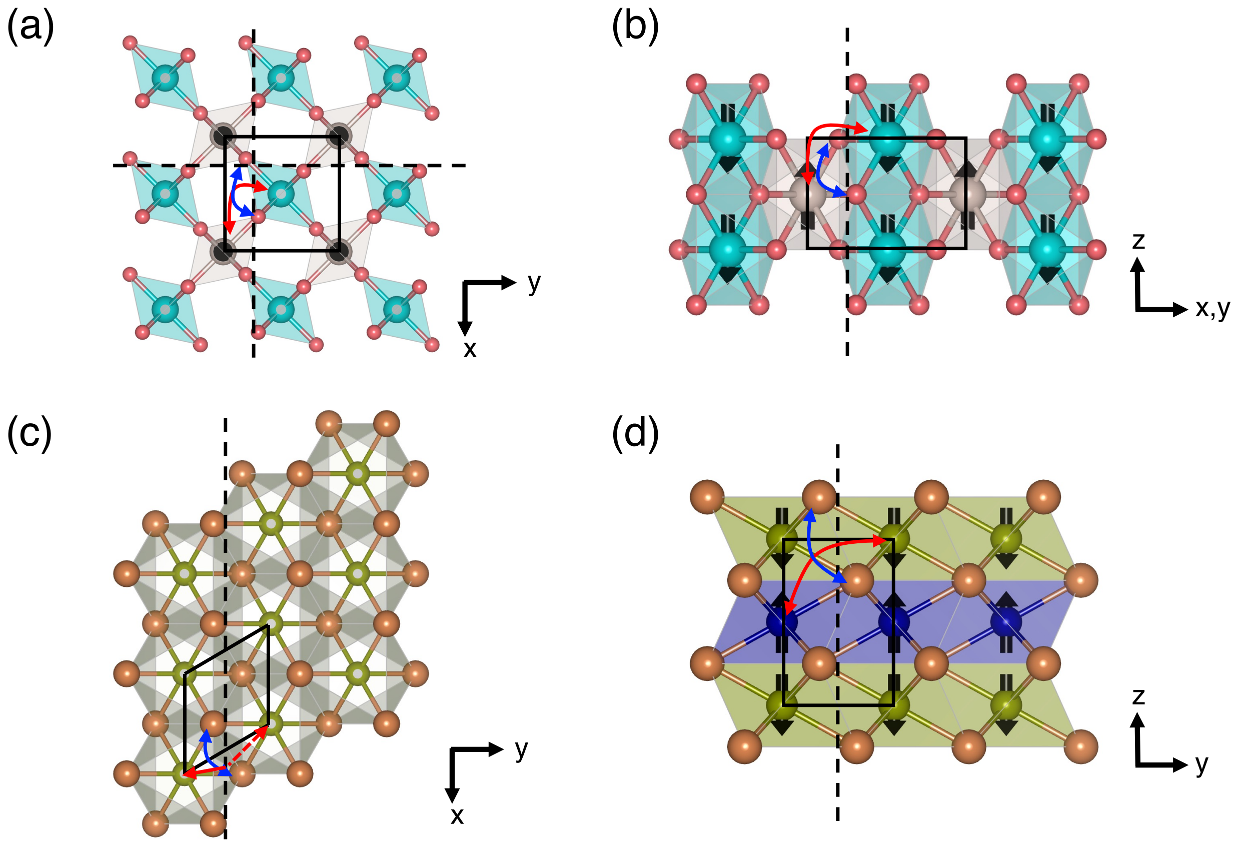

FIG. S5. Glide-reflection symmetry of RuO_2 and CrSb structures. The black box in the figure represents the unit cell, and the dashed line indicates the mirror plane. The red and blue double-headed arrows represent glide-reflection symmetry pairs. (a-b) Top and side views of the RuO_2 structure. The combination of the mirror operation ($\mathcal{M}_{x,y}$) and the half-translation operation ($\tau_{y+1/2,x+1/2} + \tau_{z+1/2}$) satisfies the glide-reflection symmetry. (c-d) Top and side views of the CrSb structure. The dotted arrow in (c) points to the atom one layer below along the z-axis. The combination of the mirror operation ($\mathcal{M}_y$) and the half-translation operation ($\tau_x + \tau_z$) satisfies the glide-reflection symmetry.

Nonsymmorphic symmetries combine a fractional lattice translational operation with another symmetry operation, such as mirror reflection or rotation. Examples include glide-reflection and screw rotation, which introduce unique constraints on the band structure by influencing degenerate electronic states. Among them, RuO_2 and CrSb exhibit glide-reflection symmetry due to their antiferromagnetic order along the z-axis, as illustrated in

Fig. S5. This arises because spins reverse their orientation under a mirror operation relative to a parallel mirror plane.

In Fig. S5, dashed lines indicate mirror planes, while red and blue double-headed arrows represent glide-reflection symmetry pairs, visually clarifying the concept of glide-reflection symmetry. The black box marks the unit cell. As shown in Figs. S5(a) and (b), glide-reflection symmetry results from the combination of the mirror operation ($\mathcal{M}_{x,y}$) and a half-translation operation ($\tau_{y+1/2,x+1/2} + \tau_{z+1/2}$). The half-translation operation essential for glide-reflection symmetry is evident in the top view of Fig. S5(a) and the side view of Fig. S5(b).

Similarly, in Figs. S5(c) and (d), glide-reflection symmetry arises from the combination of the mirror operation ($\mathcal{M}_y$) and the half-translation operation ($\tau_x + \tau_z$). The dotted arrow in Fig. S5(c) points to an atom positioned one layer below along the z-axis.

In contrast, MnTe, with its in-plane antiferromagnetic order, lacks glide-reflection symmetry. However, it retains mirror symmetry with respect to a plane perpendicular to the z-axis.

S5. MAGNETIC SYMMETRY-INDUCED USHC

A $(0\bar{1}1)$-oriented RuO_2 introduces a structural tilt, as depicted in Fig. 2(d)(main text), which enables unconventional USHC matrix elements. This tilt alters both the structural and spin directions, giving rise to USHC components. Interestingly, these components manifest identically in both Fermi sea and Fermi surface contributions, classifying them as trivial USHC. Figs. 2(e) and (f)(main text) present the calculated Fermi sea and surface contributions to the SHC for $(0\bar{1}1)$-oriented RuO_2. The values of $\sigma_{zz}^{\mathrm{odd},x}$, $\sigma_{xz}^{\mathrm{odd},z}$, and $\sigma_{zx}^{\mathrm{odd},z}$ are determined to be 0.21, 1.05, and 0.89×10^5 $(\hbar/2e)$ S/m at the Fermi level, consistent with previous findings[S2].

The trivial nature of the USHC is evidenced by the identical matrix elements obtained from both Fermi sea and surface contributions, irrespective of the tilting angle. For a structural tilt of 34.18° between the z- and y-axes, the SHC components are projected as:

$$(v \text{ or } \sigma)_{\mathrm{tilted},y} \to \cos(-34.18^\circ)(v \text{ or } \sigma)_y - \sin(-34.18^\circ)(v \text{ or } \sigma)_z,$$

demonstrating the projection of SHC components along the tilted spin direction. Using this projection, all SHC tensor elements for tilted structures can be computed analytically, removing the need for direct DFT simulations.

While this analytical framework is broadly applicable to various materials and tilt angles, its utility is constrained by the inherent reduction in spin transport efficiency. This reduction arises from the angle-dependent redistribution of conductivity components within the tilted structure, which disrupts the coherence of spin transport pathways—a critical factor for achieving high spin Hall performance. These findings highlight the trade-off between structural flexibility and transport efficiency, offering a theoretical foundation for optimizing spin Hall materials across diverse tilt geometries.

In contrast, CrSb exhibits unique bulk spin-momentum locking, as demonstrated by the spin texture in Fig. 4(e)(main text), derived from the band indicated by the arrow in Fig. 4(d)(main text). Under non-magnetic conditions, CrSb maintains the same mirror symmetry planes as RuO_2, including $(k_x = 0, k_y, k_z)$, $(k_x, k_y = 0, k_z)$, and $(k_x, k_y, k_z = 0)$. The mirror plane $(k_x, k_y = 0, k_z)$, associated with the glide-reflection symmetry $\mathcal{M}_y(\tau_x + \tau_z)$, remains intact under non-magnetic conditions, as detailed in Supplementary S4. However, magnetic symmetry breaks mirror planes such as $(k_x = 0, k_y, k_z)$ and $(k_x, k_y, k_z = 0)$, leading

to SOC-induced spin splitting within the nodal plane $k_z = 0$, as shown in the band structure in Fig. 4(d)(main text) and the in-plane spin texture in Fig. 4(f)(main text).

To evaluate the SHC tensor elements arising from Fermi surface contributions, symmetry operations are systematically applied to S_{ij}^k. Specifically, the time-reversal operation combined with a 180° rotation about the x-axis ($\mathcal{T}C_{2x}$) and the glide-reflection operation $\mathcal{M}_y(\tau_{x+1/2} + \tau_{z+1/2})$ determine the allowable SHC elements. For instance, applying these operations to S_{xy}^z yields:

$$\mathcal{T}C_{2x}S_{xy}^z \equiv \mathcal{T}[v_x(-\sigma_z)(-v_y)] = (-v_x)\sigma_z v_y \equiv -S_{xy}^z,$$
$$\mathcal{M}_y(\tau_{x+1/2} + \tau_{z+1/2})S_{xy}^z \equiv v_x(-\sigma_z)(-v_y) \equiv S_{xy}^z.$$

The C_{2x} operation preserves the parity of v_x and σ_x as even, while flipping the parity of other components to odd. Time-reversal symmetry introduces odd parity in angular momentum and velocity components, whereas the half-translation operator $(\tau_{x+1/2} + \tau_{z+1/2})$ does not alter the sign of any component. The glide-reflection operation $\mathcal{M}_y$ reverses the signs of v_y, σ_x, and σ_z. Thus, SHC tensor elements must retain even parity under all symmetry operations to be allowed. This same principle applies to CSHC elements, prohibiting all CSHC components under the imposed symmetry constraints. However, for USHC elements such as S_{yy}^y:

$$\mathcal{T}C_{2x}S_{yy}^y \equiv \mathcal{T}[(-v_y)(-\sigma_y)(-v_y)] = v_y\sigma_y v_y \equiv S_{yy}^y,$$
$$\mathcal{M}_y(\tau_{x+1/2} + \tau_{z+1/2})S_{yy}^y \equiv (-v_y)\sigma_y(-v_y) \equiv S_{yy}^y.$$

Here, $\sigma_{yy}^{\mathrm{odd},y}$ is symmetry-allowed, with equivalent components $\sigma_{xx}^{\mathrm{odd},y}$, $\sigma_{yx}^{\mathrm{odd},x}$, and $\sigma_{xy}^{\mathrm{odd},x}$ constrained by C_{3z} rotational symmetry.

Similarly, applying all symmetry operations of the MnTe system to S_{ij}^k reveals which SHC tensor elements remain. The reduced symmetry of MnTe allows for a broader set of USHC tensor elements compared to RuO_2 and CrSb. For example, MnTe's unique symmetry properties permit tensor components such as $\sigma_{xx}^{\mathrm{odd},z}$, $\sigma_{xz}^{\mathrm{odd},x}$, $\sigma_{yy}^{\mathrm{odd},z}$, $\sigma_{zx}^{\mathrm{odd},x}$, $\sigma_{zy}^{\mathrm{odd},y}$, and $\sigma_{zz}^{\mathrm{odd},z}$.

S6. k-RESOLVED BERRY-CURVATURE ANALYSIS

To elucidate the microscopic origin of the spin Hall conductivity (SHC), we analyzed the k-resolved Berry-curvature–like quantities that determine the $\mathcal{T}$-even and $\mathcal{T}$-odd components of the SHC tensor. These quantities provide a direct visualization of how crystal and magnetic symmetries govern the spatial distribution and sign compensation of the spin Hall response in RuO_2, CrSb, and MnTe. The Berry-curvature–like terms are defined as

$$\Omega_{ij}^{\mathrm{even},k}(\mathbf{k}) = -2\sum_{n\neq m}\frac{\mathrm{Im}(\langle n|\frac{1}{2}\{\hat{s}_k,\hat{v}_i\}|m\rangle\langle m|\hat{v}_j|n\rangle)}{(\epsilon_n-\epsilon_m)^2}, \tag{S1}$$

$$\Omega_{ij}^{\mathrm{odd},k}(\mathbf{k}) = -\Gamma^2\sum_{n,m}\frac{\mathrm{Re}(\langle n|\frac{1}{2}\{\hat{s}_k,\hat{v}_i\}|m\rangle\langle m|\hat{v}_j|n\rangle)}{[(E_\mathrm{F}-\epsilon_n)^2+\Gamma^2][(E_\mathrm{F}-\epsilon_m)^2+\Gamma^2]}, \tag{S2}$$

where $\hat{s}_k$, $\hat{v}_{i,j}$ denote the spin and velocity operators, and Γ is the lifetime broadening parameter. Eq. (S1) represents the $\mathcal{T}$-even "Fermi-sea" contribution that is determined purely by crystal symmetry, while Eq. (S2) gives the $\mathcal{T}$-odd "Fermi-surface" term that changes sign under spin reversal.

Figs. S6 and S7 display the k-resolved maps of $\Omega_{ij}^{\mathrm{even},k}(\mathbf{k})$ and $\Omega_{ij}^{\mathrm{odd},k}(\mathbf{k})$ for RuO_2, CrSb, and MnTe at $E_\mathrm{F}-0.5$ eV. For the $\mathcal{T}$-even channel, all three systems are symmetry-allowed only for the conventional tensor components, and the k-resolved distributions show clear non-compensated patterns—regions predominantly positive or negative—reflecting a net conventional SHC controlled by crystal symmetry. For the $\mathcal{T}$-odd channel, RuO_2 retains such non-compensated features because its $4/mmm$ symmetry still allows conventional SHC elements.

In contrast, CrSb and MnTe, where these components are symmetry-forbidden, exhibit strong sign compensation between red (+) and blue (-) regions, a hallmark of unconventional SHC emerging from magnetic-symmetry breaking and spin mixing. These k-resolved "hot-spot" maps thus demonstrate that the SHC originates from extended Fermi-surface regions rather than isolated band crossings. Fig. S8 shows k-resolved slices of $\Omega_{ij}^{\mathrm{odd},k}(\mathbf{k})$ at $k_z = -0.25, 0, 0.25$ for CrSb and MnTe. For CrSb, the unconventional SHC is mainly induced on the nodal plane ($k_z = 0$), where mirror and rotational symmetries allow finite $\mathcal{T}$-odd components. In MnTe, by contrast, the horizontal mirror $\mathcal{M}_z$ preserves the z-spin component, so finite but weaker contributions persist even away from the nodal plane. These results confirm that the symmetry of the magnetic space group—not merely the presence

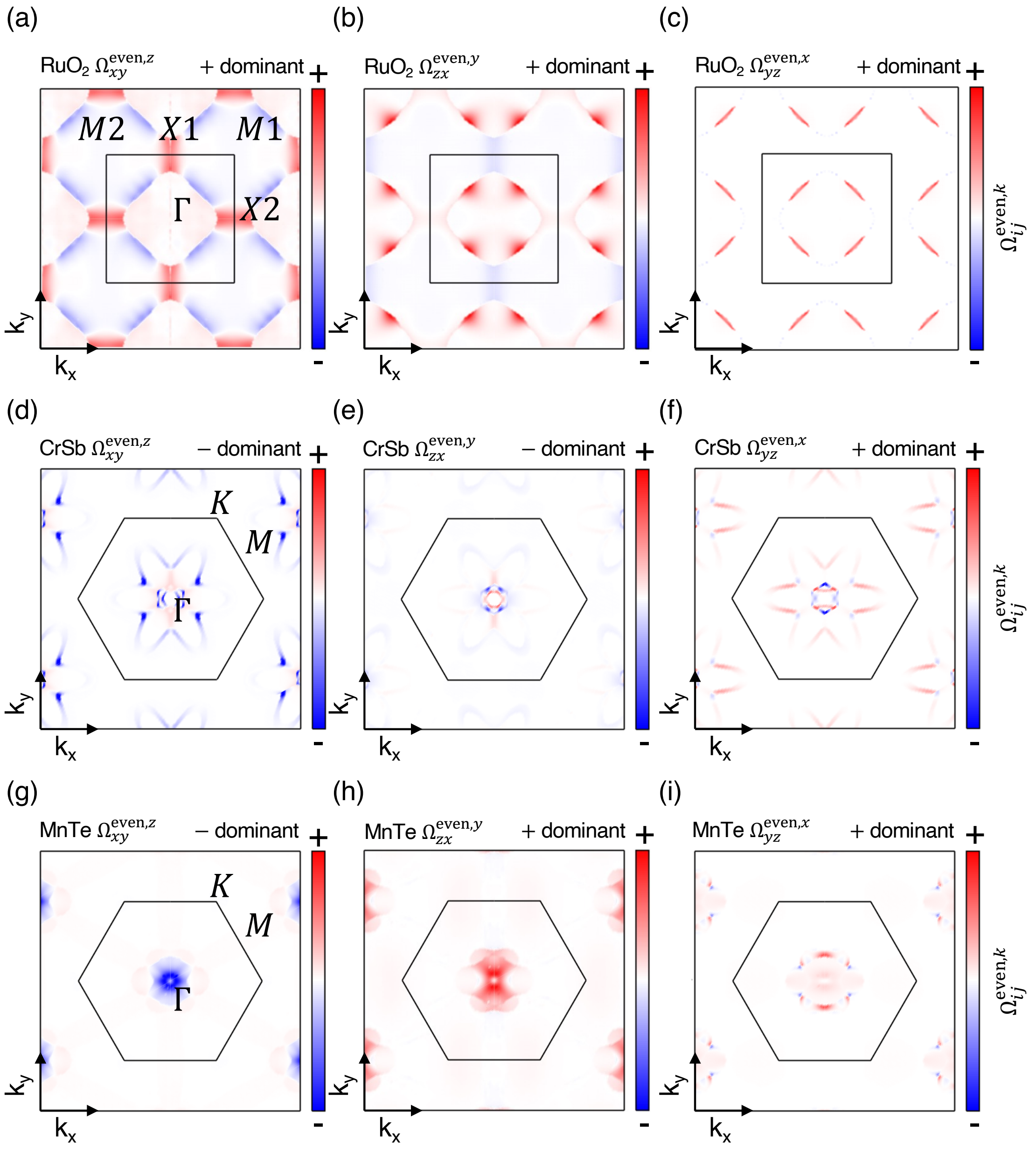

FIG. S6. k-resolved $\mathcal{T}$-even Berry-curvature–like term $\Omega_{ij}^{\mathrm{even},k}$ at $E_{\mathrm{F}}-0.5$ eV. (a–c) RuO_2, (d–f) CrSb, and (g–i) MnTe. Each column corresponds to the spin component $k = z, y, x$, respectively. The color scale represents the sign and magnitude of the $\mathcal{T}$-even Berry-curvature–like quantity $\Omega_{ij}^{\mathrm{even},k}(\mathrm{k})$ (red = positive, blue = negative; arbitrary units). Because the $\mathcal{T}$-even SHC in RuO_2, CrSb, and MnTe is symmetry-allowed only for the conventional tensor components, the k-resolved spin Berry-curvature–like term shows a clear imbalance toward either positive or negative values, indicating a net conventional response governed purely by crystal symmetry.

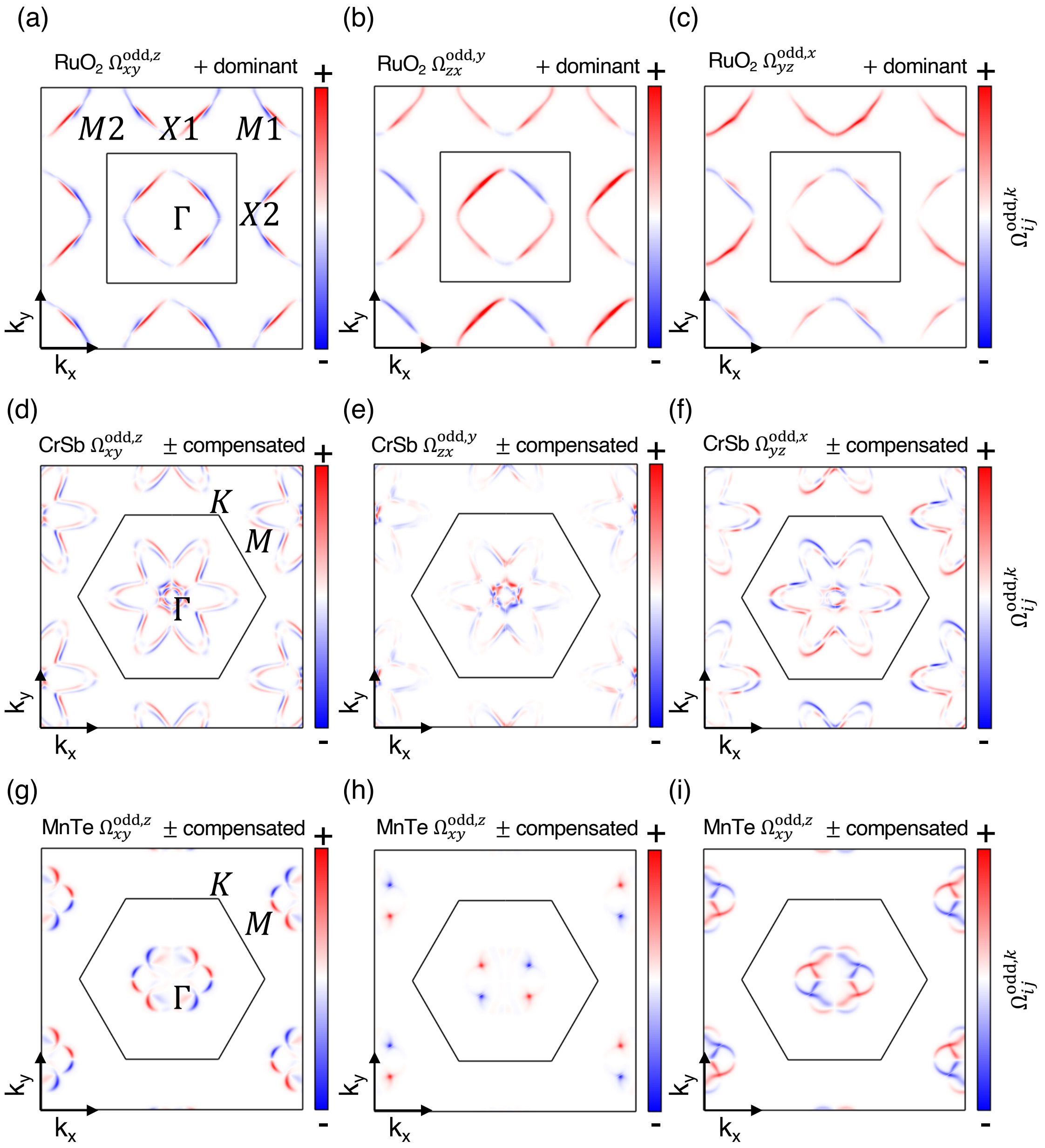

FIG. S7. k-resolved $\mathcal{T}$-odd Berry-curvature–like term $\Omega_{ij}^{\mathrm{odd},k}$ at $E_{\mathrm{F}}-0.5$ eV. (a–c) RuO_2, (d–f) CrSb, and (g–i) MnTe. Each column corresponds to the spin component $k = z, y, x$, respectively. The color scale represents the sign and magnitude of the $\mathcal{T}$-odd Berry-curvature–like quantity $\Omega_{ij}^{\mathrm{odd},k}(\mathrm{k})$ (red = positive, blue = negative; arbitrary units). For the $\mathcal{T}$-odd contribution, RuO_2 retains non-compensated Berry-curvature–like features because its symmetry still allows CSHC components, whereas in CrSb and MnTe, the absence of such symmetry-allowed terms leads to strong sign compensation of the k-resolved pattern.

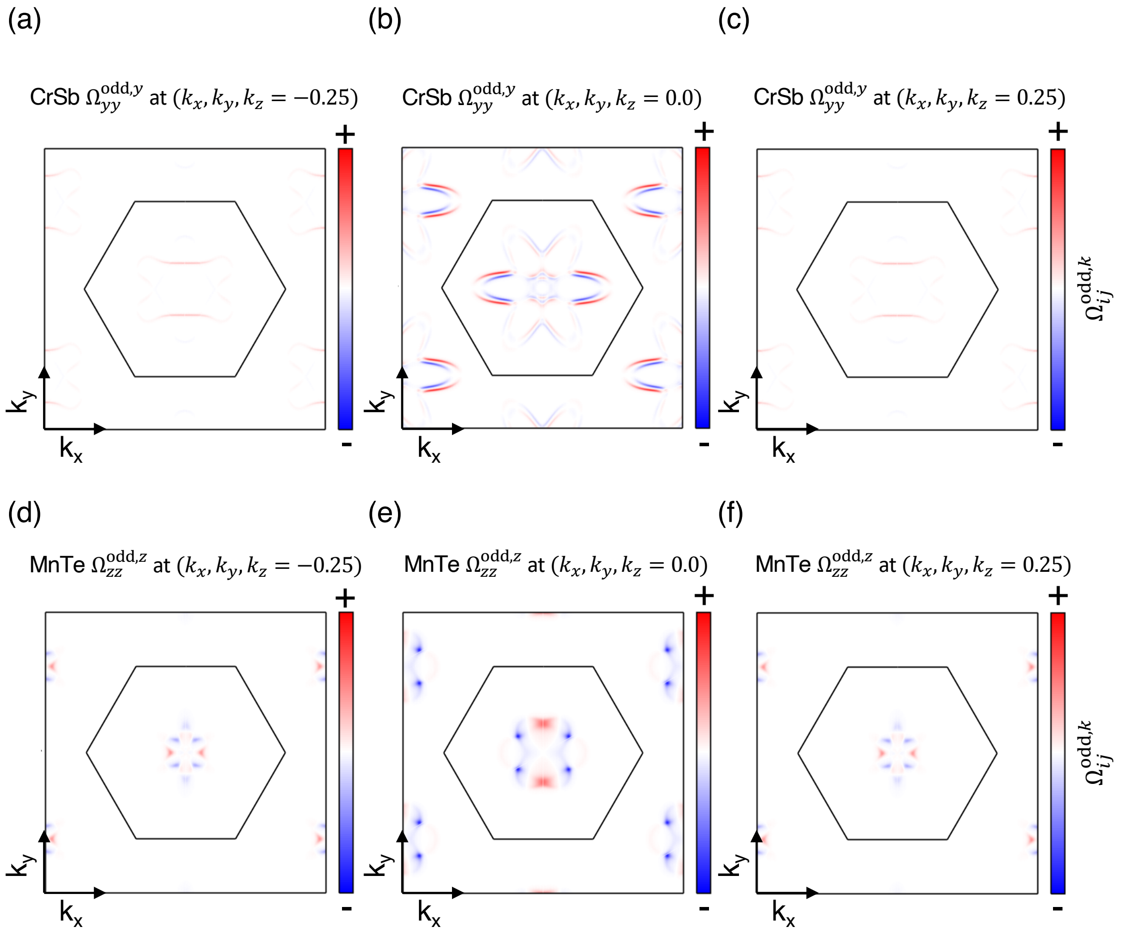

FIG. S8. k-resolved $\mathcal{T}$-odd Berry-curvature–like unconventional term $\Omega_{ij}^{\mathrm{odd},k}$ at $E_{\mathrm{F}} - 0.5$ eV with $k_z = -0.25, 0, 0.25$. (a–c) $\Omega_{yy}^{\mathrm{odd},y}$ of CrSb, and (d–f) $\Omega_{zz}^{\mathrm{odd},z}$ of MnTe. The color scale indicates the sign and magnitude of the $\mathcal{T}$-odd contribution (red = positive, blue = negative), and for $k_z = -0.25$ and $k_z = 0.25$ slices, the color range is normalized to that of the $k_z = 0$ plane for direct comparison. For CrSb, the results indicate that the USHC is mainly induced on the nodal plane, where the mirror and rotational symmetries permit finite $\mathcal{T}$-odd components. In contrast, for MnTe, the presence of the horizontal mirror symmetry $\mathcal{M}_z$ preserves the z-spin component, so that although the magnitude is smaller, finite contributions still appear even away from the nodal plane.

of SOC—determines the spatial extent and sign compensation of the Berry-curvature distribution.

S7. WANNIER FUNCTION CENTERS AND SPREADS

```
Final State
 WF centre and spread    1  (  2.313060,  2.310085, -1.384484 )     1.05093046
 WF centre and spread    2  (  0.072919, -0.208005, -0.771345 )     2.58412162
 WF centre and spread    3  (  0.593334,  0.576556,  0.359223 )     7.15159323
 WF centre and spread    4  (  1.274795, -0.465880,  0.084203 )     4.79379132
 WF centre and spread    5  ( -0.832078,  1.156046,  0.309311 )     4.86846153
 WF centre and spread    6  ( -0.461368,  1.331467,  0.039352 )     4.73488202
 WF centre and spread    7  (  1.130201, -0.838508,  0.324497 )     4.79545553
 WF centre and spread    8  (  1.910042,  1.917414,  1.461431 )     3.04784488
 WF centre and spread    9  (  2.244447,  2.244304,  1.566271 )     1.04376204
 WF centre and spread   10  (  2.283918,  2.283339,  1.438764 )     1.06937830
 WF centre and spread   11  ( -0.811734,  0.874566,  1.444867 )     1.59070643
 WF centre and spread   12  ( -0.974610,  0.996784,  1.515995 )     1.54740805
 WF centre and spread   13  (  0.880033, -0.815627,  1.441402 )     1.61863973
 WF centre and spread   14  (  0.998062, -0.971295,  1.505680 )     1.60310278
 WF centre and spread   15  (  2.268130,  2.268177,  1.567987 )     0.83196901
 WF centre and spread   16  (  2.258856,  2.257536,  1.559935 )     0.89108402
 WF centre and spread   17  (  2.276266,  2.264294,  1.498676 )     0.96410360
 WF centre and spread   18  (  2.101812,  2.101647,  1.489585 )     1.02330378
 WF centre and spread   19  (  1.045569,  1.069303,  0.026479 )     1.48453549
 WF centre and spread   20  ( -0.926513, -0.953826,  0.830004 )     7.73110341
 WF centre and spread   21  ( -0.265480,  0.108585, -0.902285 )     1.85341037
 WF centre and spread   22  (  0.179430, -0.340182, -1.152044 )     2.27845066
 WF centre and spread   23  ( -0.796719,  0.647354, -1.095238 )     1.14715746
 WF centre and spread   24  ( -0.751693, -0.941971, -0.156273 )     2.05770807
 WF centre and spread   25  (  0.517430, -0.644177, -0.909997 )     1.50109670
 WF centre and spread   26  ( -0.892664, -0.625528, -0.201618 )     2.16681745
 WF centre and spread   27  ( -0.057033, -0.054148,  0.105311 )     2.37733736
 WF centre and spread   28  (  0.012842, -0.003004,  0.022779 )     1.84522906
 WF centre and spread   29  (  0.040490, -0.004227,  0.056084 )     1.77135887
 WF centre and spread   30  (  0.010943,  0.001306, -0.007790 )     1.38587760
 WF centre and spread   31  (  0.001505,  0.032549,  0.056488 )     1.77459989
 WF centre and spread   32  (  0.002165,  0.013480, -0.008132 )     1.39528917
 WF centre and spread   33  ( -0.018655, -0.006777, -0.000622 )     0.97646712
 WF centre and spread   34  ( -1.214547, -1.171911, -0.043402 )     1.32667448
 WF centre and spread   35  (  0.038503,  0.048220, -0.017030 )     2.13558732
 WF centre and spread   36  (  0.028828,  0.021060,  0.008855 )     2.10678811
 WF centre and spread   37  ( -0.848409,  1.235885,  1.509789 )     0.82049759
 WF centre and spread   38  ( -0.756500,  1.168571,  1.477705 )     0.73221768
 WF centre and spread   39  ( -1.098910,  0.834087,  1.292966 )     0.80773686
 WF centre and spread   40  ( -1.103939,  0.839133,  1.339630 )     0.73056597
 WF centre and spread   41  ( -1.844724,  2.635962,  0.786028 )     2.11970898
 WF centre and spread   42  ( -1.548913,  2.868283,  0.701292 )     3.97046800
 WF centre and spread   43  (  1.235343, -0.849172,  1.511161 )     0.82416749
 WF centre and spread   44  (  1.165227, -0.751670,  1.469393 )     0.76585111
 WF centre and spread   45  (  2.547920, -1.939606,  0.966078 )     1.93572702
 WF centre and spread   46  (  2.404447, -2.026111,  1.302247 )     1.66221877
 WF centre and spread   47  (  0.833538, -1.099290,  1.289113 )     0.81673076
 WF centre and spread   48  (  0.846191, -1.110663,  1.334745 )     0.73962177
 WF centre and spread   49  (  1.393804,  1.393351,  0.078988 )     1.15170635
 WF centre and spread   50  (  1.497340,  1.496662, -0.340017 )     1.57834272
 WF centre and spread   51  (  1.706498,  1.293203, -0.063553 )     0.61444759
 WF centre and spread   52  (  1.686179,  1.297892,  0.070737 )     0.65857449
 WF centre and spread   53  (  1.297593,  1.707894, -0.063429 )     0.62136628
 WF centre and spread   54  (  1.295580,  1.688964,  0.071202 )     0.65391713
 WF centre and spread   55  ( -1.477522, -1.477287, -0.117723 )     1.26052694
 WF centre and spread   56  ( -1.479445, -1.480622, -0.096401 )     1.55455397
 WF centre and spread   57  ( -1.463546, -1.072389,  0.006487 )     0.69955966
 WF centre and spread   58  (  0.184394,  0.179392, -0.031078 )     0.99577032
 WF centre and spread   59  ( -1.072852, -1.462112,  0.006057 )     0.70667436
 WF centre and spread   60  (  1.082110,  1.109014,  0.007976 )     1.18319009
 Sum of centres and spreads ( 22.961888, 22.958378, 26.572311 )   110.13016880

        Spreads (Ang^2)       Omega I      =    70.231216463
       ================       Omega D      =     1.556234339
                              Omega OD     =    38.342718002
   Final Spread (Ang^2)       Omega Total  =   110.130168805
```

FIG. S9. Final Wannier function centers and spreads for RuO_2. The total spread is 110.13 Å^2 confirming well-converged spinor Wannier functions used in SHC analysis.

```
Final State
 WF centre and spread    1  ( -0.540039, -1.062436,  0.407521 )     4.97022856
 WF centre and spread    2  ( -0.732803,  0.045146, -1.134613 )     1.73511341
 WF centre and spread    3  ( -1.238830, -1.205986,  0.934539 )     2.20230204
 WF centre and spread    4  ( -0.716115, -1.127688,  0.850037 )     6.24870487
 WF centre and spread    5  (  1.266410,  0.967666, -0.696523 )     1.21059865
 WF centre and spread    6  (  1.277969,  0.987182, -0.751960 )     1.49034948
 WF centre and spread    7  ( -1.505868, -1.100935,  0.780998 )     0.81081279
 WF centre and spread    8  ( -1.447353, -1.089109,  0.782413 )     0.75843126
 WF centre and spread    9  (  1.548435,  1.380543, -0.613846 )     1.34555000
 WF centre and spread   10  (  1.587409,  1.384972, -0.605919 )     1.09396346
 WF centre and spread   11  (  1.707539,  1.037399, -1.630062 )     2.52692588
 WF centre and spread   12  (  1.642963,  0.983627, -1.142711 )     1.07271401
 WF centre and spread   13  (  1.287979,  1.425569, -1.156117 )     2.06043781
 WF centre and spread   14  (  1.310837,  1.446316, -0.919136 )     1.16890481
 WF centre and spread   15  (  1.408543,  1.230532, -1.238905 )     3.53867620
 WF centre and spread   16  (  2.093748,  2.525273, -0.178308 )     1.34691006
 WF centre and spread   17  ( -1.281547,  2.267835, -1.227569 )     3.20631228
 WF centre and spread   18  ( -1.664890,  2.451028, -0.518504 )     2.66026820
 WF centre and spread   19  ( -1.330312, -1.243405,  0.590929 )     1.40995474
 WF centre and spread   20  ( -0.959951, -0.863759,  0.477831 )     2.64992294
 WF centre and spread   21  ( -0.863265,  0.001902, -1.369385 )     1.02729722
 WF centre and spread   22  ( -1.498492, -1.022999,  1.450992 )     3.35870743
 WF centre and spread   23  ( -0.343109, -0.087247, -0.376093 )     1.47720405
 WF centre and spread   24  ( -0.384424, -0.220789,  0.060845 )     1.48568803
 WF centre and spread   25  ( -1.456442, -1.217216,  0.708034 )     1.32154364
 WF centre and spread   26  (  1.109038,  1.139986, -1.265797 )     8.37038893
 WF centre and spread   27  ( -0.018678,  0.012078, -0.073434 )     1.77662887
 WF centre and spread   28  ( -0.001508, -0.003068, -0.046202 )     1.37909449
 WF centre and spread   29  ( -0.012122,  0.023710, -0.012450 )     1.65714217
 WF centre and spread   30  ( -0.013667,  0.006819, -0.021830 )     1.43379735
 WF centre and spread   31  (  0.007254, -0.077359, -0.008808 )     1.62007881
 WF centre and spread   32  ( -0.007254, -0.042894, -0.010525 )     1.48900697
 WF centre and spread   33  (  0.011602, -0.002872,  0.001049 )     1.17319431
 WF centre and spread   34  ( -0.007963,  0.005879, -0.004839 )     1.08001130
 WF centre and spread   35  ( -0.072494,  0.062816, -0.045381 )     2.32975286
 WF centre and spread   36  ( -0.049942,  0.055013, -0.030803 )     1.89066801
 WF centre and spread   37  ( -1.189507,  1.906275,  0.505400 )     1.30739275
 WF centre and spread   38  ( -1.209225,  1.922142,  0.486969 )     1.23435725
 WF centre and spread   39  ( -0.964347,  1.308127,  0.724368 )     0.69412518
 WF centre and spread   40  ( -0.947927,  1.302303,  0.732684 )     0.66474211
 WF centre and spread   41  ( -0.705287,  1.702078,  0.715317 )     0.88144073
 WF centre and spread   42  ( -0.694173,  1.700863,  0.723926 )     0.77992202
 WF centre and spread   43  (  2.128081, -0.838108,  2.412510 )     1.19698879
 WF centre and spread   44  (  1.174953,  0.086595,  1.619172 )     0.90870664
 WF centre and spread   45  (  2.031575,  0.471163,  0.489129 )     6.57611221
 WF centre and spread   46  (  1.880261,  0.572530,  0.760141 )     6.98905243
 WF centre and spread   47  (  1.117525, -0.701894,  1.187816 )     6.21678361
 WF centre and spread   48  (  1.064332, -0.576673,  0.903053 )     4.88730886
 WF centre and spread   49  (  0.597780,  0.040535,  1.791344 )     0.89643620
 WF centre and spread   50  (  0.587997,  0.104541,  1.778262 )     0.81790562
 WF centre and spread   51  (  0.316938, -0.061452,  2.006743 )     4.46478597
 WF centre and spread   52  ( -0.472777, -0.264832,  2.164109 )     5.23269492
 WF centre and spread   53  (  1.294415, -0.278776,  2.058211 )     1.60060659
 WF centre and spread   54  (  1.154204, -0.162530,  2.025312 )     1.31449234
 WF centre and spread   55  (  0.975922,  0.231306,  1.571600 )     0.88827360
 WF centre and spread   56  (  2.085905, -0.783047,  2.297128 )     1.44499679
 WF centre and spread   57  (  2.279794,  2.741368,  0.002581 )     0.92785270
 WF centre and spread   58  (  2.347330,  2.732317,  0.009251 )     0.88672384
 WF centre and spread   59  (  2.213176,  2.722501,  0.019051 )     1.04095292
 WF centre and spread   60  (  2.149233,  2.746388,  0.097425 )     1.01609357
 Sum of centres and spreads ( 19.328835, 27.697251, 19.046969 )   129.24603354

        Spreads (Ang^2)       Omega I      =    72.278985022
       ================       Omega D      =     2.000159347
                              Omega OD     =    54.966888814
    Final Spread (Ang^2)      Omega Total  =   129.246033183
```

FIG. S10. Final Wannier function centers and spreads for tilted RuO_2. The total spread is 129.25 $Å^2$ confirming well-converged spinor Wannier functions used in SHC analysis.

```
 Final State
  WF centre and spread    1  ( -0.008552, -0.001116,  2.660570 )     3.42194495
  WF centre and spread    2  (  0.001371, -0.043793,  2.659787 )     3.70417701
  WF centre and spread    3  (  0.000132, -0.000072,  2.661975 )     6.30702932
  WF centre and spread    4  (  0.000062,  0.000014,  2.662864 )     6.42449353
  WF centre and spread    5  (  0.009171, -0.000003,  2.661248 )     4.87424305
  WF centre and spread    6  ( -0.001521, -0.000022,  2.661232 )     5.27675834
  WF centre and spread    7  (  0.000009,  0.001176,  2.661233 )     4.89004207
  WF centre and spread    8  (  0.000006,  0.048445,  2.660240 )     5.29635129
  WF centre and spread    9  ( -0.000070, -0.000011,  2.661227 )     0.58760977
  WF centre and spread   10  (  0.000016, -0.000490,  2.661194 )     0.68016738
  WF centre and spread   11  ( -0.000012, -0.000001,  2.661247 )     0.82110298
  WF centre and spread   12  ( -0.000035, -0.000001,  2.661247 )     1.03447417
  WF centre and spread   13  ( -0.000000,  0.000001,  2.661253 )     0.82163582
  WF centre and spread   14  (  0.000003, -0.000119,  2.661814 )     1.03533339
  WF centre and spread   15  ( -0.000456, -0.000057,  2.661250 )     0.78145100
  WF centre and spread   16  (  0.000107, -0.003397,  2.661650 )     1.02817909
  WF centre and spread   17  ( -0.000002,  0.000001,  2.661246 )     0.78129975
  WF centre and spread   18  ( -0.000003,  0.000005,  2.661248 )     1.02798261
  WF centre and spread   19  (  0.013223, -0.008237, -0.000006 )     3.70522624
  WF centre and spread   20  (  0.001997,  0.003904,  0.001861 )     3.42162275
  WF centre and spread   21  ( -0.000066, -0.000009, -0.000005 )     6.42342932
  WF centre and spread   22  (  0.000031, -0.000470, -0.001979 )     6.30627794
  WF centre and spread   23  ( -0.014622, -0.000004,  0.000000 )     5.27643587
  WF centre and spread   24  ( -0.002134, -0.000022, -0.000008 )     4.87453630
  WF centre and spread   25  ( -0.000003,  0.009114,  0.000190 )     5.29950927
  WF centre and spread   26  (  0.000002, -0.004267, -0.000043 )     4.89016812
  WF centre and spread   27  (  0.000148, -0.000092,  0.000000 )     0.68020156
  WF centre and spread   28  (  0.000017,  0.000026,  0.000051 )     0.58755961
  WF centre and spread   29  (  0.000024, -0.000001,  0.000001 )     1.03466731
  WF centre and spread   30  ( -0.000009, -0.000003, -0.000001 )     0.82110023
  WF centre and spread   31  (  0.000021, -0.000022, -0.000108 )     1.03552452
  WF centre and spread   32  (  0.000001,  0.000045,  0.000018 )     0.82163427
  WF centre and spread   33  (  0.001025, -0.000640, -0.000076 )     1.02806671
  WF centre and spread   34  (  0.000106,  0.000219,  0.000011 )     0.78134393
  WF centre and spread   35  (  0.000001, -0.000000, -0.000000 )     1.02795466
  WF centre and spread   36  ( -0.000001,  0.000001, -0.000000 )     0.78119681
  WF centre and spread   37  ( -0.000004,  2.412115,  3.931155 )     3.11705699
  WF centre and spread   38  ( -0.000005,  2.412127,  4.052862 )     3.11695096
  WF centre and spread   39  ( -0.000023,  2.213046,  3.944727 )     2.66257551
  WF centre and spread   40  ( -0.000013,  2.213068,  4.038954 )     2.66214138
  WF centre and spread   41  (  0.000006,  2.615184,  3.944526 )     2.66194250
  WF centre and spread   42  ( -0.000002,  2.615250,  4.039304 )     2.66239221
  WF centre and spread   43  (  2.089007,  1.206154,  1.391325 )     3.11707202
  WF centre and spread   44  (  2.089007,  1.206165,  1.269647 )     3.11698105
  WF centre and spread   45  (  2.088987,  1.405207,  1.377794 )     2.66282166
  WF centre and spread   46  (  2.088997,  1.405147,  1.283353 )     2.66331415
  WF centre and spread   47  (  2.089019,  1.003141,  1.377937 )     2.66170678
  WF centre and spread   48  (  2.089011,  1.003285,  1.283360 )     2.66130508
  Sum of centres and spreads ( 12.533973, 21.709990, 79.837376 )   131.35699124

         Spreads (Ang^2)       Omega I      =    99.391628816
       ================        Omega D      =     0.019780723
                               Omega OD     =    31.945581469
    Final Spread (Ang^2)       Omega Total  =   131.356991007
```

FIG. S11. Final Wannier function centers and spreads for CrSb. The total spread is 131.36 Å^2 confirming well-converged spinor Wannier functions used in SHC analysis.

```
 Final State
  WF centre and spread    1  (  0.004095,  0.018146, -0.094557 )     3.48181466
  WF centre and spread    2  ( -0.014968,  0.014967, -0.062576 )     3.47977305
  WF centre and spread    3  (  0.000071,  0.000020,  0.103821 )     6.93341156
  WF centre and spread    4  (  0.000185,  0.000181,  0.068632 )     6.94471853
  WF centre and spread    5  ( -0.004526, -0.000126, -0.000160 )     5.95988883
  WF centre and spread    6  (  0.016580, -0.000100, -0.000116 )     5.94834889
  WF centre and spread    7  (  0.000036, -0.019866, -0.000509 )     5.95830111
  WF centre and spread    8  (  0.000135, -0.016645, -0.000318 )     5.96704808
  WF centre and spread    9  (  0.000064,  0.000318, -0.005865 )     0.72679119
  WF centre and spread   10  ( -0.000255,  0.000278, -0.003998 )     0.72671441
  WF centre and spread   11  ( -0.000002,  0.000004,  0.000017 )     0.82373065
  WF centre and spread   12  ( -0.000002,  0.000003,  0.000015 )     0.82368011
  WF centre and spread   13  (  0.000000,  0.000006,  0.000176 )     0.82391598
  WF centre and spread   14  ( -0.000008,  0.000009,  0.000140 )     0.82372953
  WF centre and spread   15  (  0.000217,  0.001152,  0.000153 )     0.81597961
  WF centre and spread   16  ( -0.000917,  0.000961,  0.000121 )     0.81690461
  WF centre and spread   17  ( -0.000002,  0.000012, -0.000017 )     0.81693185
  WF centre and spread   18  ( -0.000008,  0.000007, -0.000014 )     0.81597343
  WF centre and spread   19  (  0.008626,  0.008599,  3.303898 )     3.48117801
  WF centre and spread   20  (  0.035316, -0.003005,  3.323157 )     3.47931731
  WF centre and spread   21  (  0.000037,  0.000205,  3.463682 )     6.93910022
  WF centre and spread   22  ( -0.000010, -0.000061,  3.442552 )     6.94633205
  WF centre and spread   23  ( -0.009576,  0.000153,  3.379918 )     5.95899675
  WF centre and spread   24  ( -0.039093,  0.000114,  3.379943 )     5.94584269
  WF centre and spread   25  (  0.000016, -0.009653,  3.380080 )     5.95820193
  WF centre and spread   26  ( -0.000050,  0.003403,  3.379920 )     5.96742793
  WF centre and spread   27  (  0.000170,  0.000158,  3.375333 )     0.72656238
  WF centre and spread   28  (  0.000620, -0.000053,  3.376449 )     0.72651478
  WF centre and spread   29  (  0.000002, -0.000001,  3.380083 )     0.82351968
  WF centre and spread   30  ( -0.000006, -0.000002,  3.380085 )     0.82338897
  WF centre and spread   31  (  0.000020,  0.000021,  3.380018 )     0.82374058
  WF centre and spread   32  (  0.000033,  0.000006,  3.380096 )     0.82343725
  WF centre and spread   33  (  0.000609,  0.000526,  3.379984 )     0.81571952
  WF centre and spread   34  (  0.002207, -0.000164,  3.380068 )     0.81655681
  WF centre and spread   35  ( -0.000001, -0.000010,  3.380053 )     0.81664083
  WF centre and spread   36  (  0.000002, -0.000006,  3.380056 )     0.81560747
  WF centre and spread   37  (  2.111715,  1.222996,  1.689691 )     3.10691304
  WF centre and spread   38  (  2.111714,  1.215548,  1.689639 )     3.10553176
  WF centre and spread   39  (  2.111704,  1.336736,  1.689911 )     2.69932571
  WF centre and spread   40  (  2.111699,  1.336876,  1.689972 )     2.70222043
  WF centre and spread   41  (  2.111734,  1.098904,  1.689849 )     2.70483839
  WF centre and spread   42  (  2.111747,  1.102178,  1.690045 )     2.69743627
  WF centre and spread   43  ( -0.000003,  2.434594,  5.069635 )     3.10672545
  WF centre and spread   44  ( -0.000003,  2.442050,  5.069923 )     3.10549799
  WF centre and spread   45  ( -0.000014,  2.320942,  5.069941 )     2.70046899
  WF centre and spread   46  ( -0.000020,  2.320756,  5.069939 )     2.70267254
  WF centre and spread   47  (  0.000017,  2.558638,  5.070025 )     2.70439676
  WF centre and spread   48  (  0.000027,  2.555401,  5.069928 )     2.69719873
  Sum of centres and spreads ( 12.669934, 21.945175,101.408816 )   139.40896733

         Spreads (Ang^2)       Omega I      =   106.158005460
        ================       Omega D      =     0.020052959
                               Omega OD     =    33.230907445
    Final Spread (Ang^2)       Omega Total  =   139.408965864
```

FIG. S12. Final Wannier function centers and spreads for MnTe. The total spread is 139.41 Å^2 confirming well-converged spinor Wannier functions used in SHC analysis.

* Corresponding author. email: physicsksh@gmail.com

† Corresponding author. email: ykkwon@khu.ac.kr

[S1] R. González-Hernández, L. Šmejkal, K. Vỳborny̌, Y. Yahagi, J. Sinova, T. Jungwirth, J. Železny̌, Efficient electrical spin splitter based on nonrelativistic collinear antiferromagnetism, *Physical Review Letters* **2021**, *126*, 12 127701.

[S2] A. Bose, N. J. Schreiber, R. Jain, D.-F. Shao, H. P. Nair, J. Sun, X. S. Zhang, D. A. Muller, E. Y. Tsymbal, D. G. Schlom, et al., Tilted spin current generated by the collinear antiferromagnet ruthenium dioxide, *Nature Electronics* **2022**, *5*, 5 267.

[S3] T. Berlijn, P. C. Snijders, O. Delaire, H.-D. Zhou, T. A. Maier, H.-B. Cao, S.-X. Chi, M. Matsuda, Y. Wang, M. R. Koehler, et al., Itinerant antiferromagnetism in RuO_2, *Physical Review Letters* **2017**, *118*, 7 077201.

[S4] Z. Zhu, J. Strempfer, R. Rao, C. Occhialini, J. Pelliciari, Y. Choi, T. Kawaguchi, H. You, J. Mitchell, Y. Shao-Horn, et al., Anomalous antiferromagnetism in metallic RuO_2 determined by resonant X-ray scattering, *Physical Review Letters* **2019**, *122*, 1 017202.

[S5] B. Z. Gregory, J. Strempfer, D. Weinstock, J. P. Ruf, Y. Sun, H. Nair, N. J. Schreiber, D. G. Schlom, K. M. Shen, A. Singer, Strain-induced orbital-energy shift in antiferromagnetic RuO_2 revealed by resonant elastic x-ray scattering, *Physical Review B* **2022**, *106*, 19 195135.

[S6] O. Fedchenko, J. Minár, A. Akashdeep, S. W. D'Souza, D. Vasilyev, O. Tkach, L. Odenbreit, Q. Nguyen, D. Kutnyakhov, N. Wind, et al., Observation of time-reversal symmetry breaking in the band structure of altermagnetic RuO_2, *Science Advances* **2024**, *10*, 5 eadj4883.

[S7] M. Hiraishi, H. Okabe, A. Koda, R. Kadono, T. Muroi, D. Hirai, Z. Hiroi, Nonmagnetic Ground State in RuO_2 Revealed by Muon Spin Rotation, *Physical Review Letters* **2024**, *132*, 16 166702.

[S8] P. Keßler, L. Garcia-Gassull, A. Suter, T. Prokscha, Z. Salman, D. Khalyavin, P. Manuel, F. Orlandi, I. I. Mazin, R. Valentí, et al., Absence of magnetic order in RuO_2: insights from μ SR spectroscopy and neutron diffraction, *npj Spintronics* **2024**, *2*, 1 50.

[S9] M. Wenzel, E. Uykur, S. Rößler, M. Schmidt, O. Janson, A. Tiwari, M. Dressel, A. A. Tsirlin, Fermi-liquid behavior of non-altermagnetic RuO_2, *arXiv preprint arXiv:2407.11148* **2024**.

[S10] J. Liu, J. Zhan, T. Li, J. Liu, S. Cheng, Y. Shi, L. Deng, M. Zhang, C. Li, J. Ding, et al.,

Absence of Altermagnetic Spin Splitting Character in Rutile Oxide RuO_2, *Physical Review Letters* **2024**, *133*, 17 176401.

[S11] A. Smolyanyuk, I. I. Mazin, L. Garcia-Gassull, R. Valentí, Fragility of the magnetic order in the prototypical altermagnet RuO_2, *Physical Review B* **2024**, *109*, 13 134424.

[S12] S. Reimers, L. Odenbreit, L. Šmejkal, V. N. Strocov, P. Constantinou, A. B. Hellenes, R. Jaeschke Ubiergo, W. H. Campos, V. K. Bharadwaj, A. Chakraborty, et al., Direct observation of altermagnetic band splitting in CrSb thin films, *Nature Communications* **2024**, *15*, 1 2116.

[S13] S. Lee, S. Lee, S. Jung, J. Jung, D. Kim, Y. Lee, B. Seok, J. Kim, B. G. Park, L. Šmejkal, et al., Broken kramers degeneracy in altermagnetic mnte, *Physical Review Letters* **2024**, *132*, 3 036702.
